# Conserved RXLR Effector Genes of *Phytophthora infestans* Expressed at the Early Stage of Potato Infection Are Suppressive to Host Defense

**DOI:** 10.3389/fpls.2017.02155

**Published:** 2017-12-19

**Authors:** Junliang Yin, Biao Gu, Guiyan Huang, Yuee Tian, Junli Quan, Hannele Lindqvist-Kreuze, Weixing Shan

**Affiliations:** ^1^College of Plant Protection, Northwest A&F University, Xianyang, China; ^2^State Key Laboratory of Crop Stress Biology for Arid Areas, Northwest A&F University, Xianyang, China; ^3^College of Life Sciences, Northwest A&F University, Xianyang, China; ^4^International Potato Center, Lima, Peru

**Keywords:** potato late blight, *Phytophthora infestans*, RXLR effectors, sequence polymorphism, durable resistance, resistance breeding

## Abstract

Late blight has been the most devastating potato disease worldwide. The causal agent, *Phytophthora infestans*, is notorious for its capability to rapidly overcome host resistance. Changes in the expression pattern and the encoded protein sequences of effector genes in the pathogen are responsible for the loss of host resistance. Among numerous effector genes, the class of RXLR effector genes is well-known in mediating host genotype-specific resistance. We therefore performed deep sequencing of five genetically diverse *P. infestans* strains using *in planta* materials infected with zoospores (12 h post inoculation) and focused on the identification of RXLR effector genes that are conserved in coding sequences, are highly expressed in early stages of plant infection, and have defense suppression activities. In all, 245 RXLR effector genes were expressed in five transcriptomes, with 108 being co-expressed in all five strains, 47 of them comparatively highly expressed. Taking sequence polymorphism into consideration, 18 candidate core RXLR effectors that were conserved in sequence and with higher *in planta* expression levels were selected for further study. *Agrobacterium tumefaciens*-mediated transient expression of the selected effector genes in *Nicotiana benthamiana* and potato demonstrated their potential virulence function, as shown by suppression of PAMP-triggered immunity (PTI) or/and effector-triggered immunity (ETI). The identified collection of core RXLR effectors will be useful in the search for potential durable late blight resistance genes. Analysis of 10 known *Avr* RXLR genes revealed that the resistance genes *R2*, *Rpi*-*blb2*, *Rpi*-*vnt1*, *Rpi*-*Smira1*, and *Rpi*-*Smira2* may be effective in potato cultivars. Analysis of 8 *SFI* (Suppressor of early Flg22-induced Immune response) RXLR effector genes showed that *SFI2, SFI3*, and *SFI4* were highly expressed in all examined strains, suggesting their potentially important function in early stages of pathogen infection.

## Introduction

Potato (*Solanum tuberosum* L.) is the world’s most important non-grain food crop and is central to global food security (Potato Genome Sequencing Consortium, 2011). However, its safe production is seriously threatened by late blight, a disease caused by *Phytophthora infestans*, the most destructive pathogen of potato (Yoshida et al., 2013). Historically, as the Irish potato famine agent, *P*. *infestans* has had a tremendous effect on human history, resulting in famine and population displacement (Haas et al., 2009). Nowadays, annual worldwide potato crop losses due to late blight are still huge, conservatively estimated at $6.7 billion (Nowicki et al., 2011). Although significant efforts have been made to control late blight disease, this pathogen is still a tremendous challenge for sustainable production of potato (Fry et al., 2015).

It is widely accepted that planting resistant cultivars is one of the most effective, economical, and environmentally friendly strategies to control *P*. *infestans* (Rodewald and Trognitz, 2013). Field-deployed resistant cultivars contain resistance (*R*) genes that can recognize *P*. *infestans* avirulence (*Avr*) RXLR effector genes and activate host defense responses to constrain disease development. However, some resistant cultivars have been defeated in a single season because the targets of potato *R* genes, *Avr* RXLR effector genes, evolve rapidly through present and absent variation (PAV), insertion and deletion (Indel), point mutations (SNPs), and gene silencing to avoid interaction with *R* genes (Raffaele et al., 2010; Vleeshouwers and Oliver, 2014). Due to the ability of *P*. *infestans* to rapidly overcome *R* genes, this pathogen is considered an “*R* gene destroyer” by phytopathologists and breeders (Haas et al., 2009; Fry et al., 2015). In order to effectively control late blight, plant breeders need to adopt new strategies and techniques in potato *R* gene identification, introgression, functional characterization, and field deployment (Vleeshouwers and Oliver, 2014).

Research over the last 15 years has led to an increasingly clear understanding of RXLR effector genes. Since Shan et al. (2004) cloned the first *P*. *sojae Avr* gene *Avr1b*, three other oomycete *Avr* genes have been cloned (Allen et al., 2004; Armstrong et al., 2005; Rehmany et al., 2005). Sequence alignment of the four AVR proteins found conservation motifs (RXLR and dEER) at their N terminals (Rehmany et al., 2005). These two motifs provide a powerful bioinformatic tool to identify the effector reservoir in oomycete pathogens, and has led to the prediction of up to 563 RXLR effector genes in the *P*. *infestans* genome (Haas et al., 2009) and 385 in the *P*. *sojae* genome (Jiang et al., 2008). Function analysis of those RXLR effectors indicated that they were secreted from the pathogen onto host cell surfaces or into host cells to defeat plant defenses by disturbing the host’s innate immune system (Wang et al., 2011). The precise virulence roles of RXLR effector genes such as *PiAvrblb1* (Champouret et al., 2009), *PiAvr3a* (Bos et al., 2010), *PiAvrblb2* (Bozkurt et al., 2011), and *PiAvr2* (Saunders et al., 2012) have been dissected; the PTI supression function of eight SFI (Suppressor of early Flg22-induced Immune response) RXLR effectors, SFI1, SFI2, SFI3, SFI4, SFI5, SFI6, SFI7, and SFI8, have been preliminary analyzed (Zheng et al., 2014); they play critical role in host–pathogen interaction, especially during the infection and colonization stages (Cooke et al., 2012). However, apart from virulence roles, RXLR effectors also play avirulence roles when potato cultivars contain cognate *R* genes (Birch et al., 2008). The underlying reason for this is that co-evolution pushes the surviving hosts to evolve *R* genes to recognize cognate effectors and trigger the localized programed cell death called hypersensitive response (HR) (Oliva et al., 2015). To date, the ten known *P*. *infestans Avr* genes, *Avr1*, *Avr2*, *Avr3a*, *Avr3b*, *Avr4*, *Avrblb1*, *Avrblb2*, *Avrvnt1*, *AvrSmira1*, and *AvrSmira2*, are all RXLR effector genes (Armstrong et al., 2005; van Poppel et al., 2008; Champouret et al., 2009; Oh et al., 2009; Gilroy et al., 2011; Vleeshouwers et al., 2011). With our increasing understanding to the function of RXLR genes, those effectors are emerging as tools in modern potato resistance breeding to accelerate and improve the identification, functional characterization, and deployment of *R* genes (Vleeshouwers and Oliver, 2014).

Recently, Dangl et al. (2013) reviewed the principle of “core effectors” and proposed an improved practice to breed durable resistance using genomic strategies that start with identification of core effectors. In fact, identification of core effectors had already been conducted by Bart et al. (2012). They used a genome sequencing strategy to search for conserved effector genes in the bacterial pathogen *Xanthomonas axonopodis* pv. *manihotis* and found a set of conserved effectors (core effectors), which now serve as targets to define the *R* genes they activate in wild species of *Manihot*, and may potentially do the same in other related plants in the *Euphorbiaceae*. Although core effectors were considered ideal targets for deploy durable resistance, *P*. *infestans* core RXLR effectors are largely unknown currently, which limits our ability to deploy durable potato *R* genes. Thus, in the search for durable potato *R* genes and long-lasting control of late blight, it is urgent to identify the core RXLR effector set in *P*. *infestans*.

Considering that (1) effector genes with important functions in pathogen virulence are sequence-conserved and widely distributed in populations (Bart et al., 2012; Dangl et al., 2013) and, (2) all known *P*. *infestans Avr* effector genes are *in planta* induced RXLR effector genes (Cooke et al., 2012), in this study we utilized a next-generation transcriptome deep sequencing strategy to identify potentially conserved core RXLR effector genes in *P*. *infestans*, primarily based on their (1) conserved sequences among diverse strains, (2) high levels of expression at the early infection stage, and (3) potentially essential functions for pathogenesis. This resulted in the identification of 18 conserved candidate Core RXLR Effectors (CRE). Transient expression of selected CREs in *Nicotiana benthamiana* suggested that these effectors contribute to the virulence of *P*. *infestans* by enhancing pathogen colonization and expansion. Further analysis revealed that these CREs defeated the host defense response by suppressing plant PTI and ETI in both *N*. *benthamiana* and potato. The identified collection of CREs is a valuable resource and they can be used as tools to search for potential durable late blight *R* genes from potato germplasms and related *Solanum* species in modern breeding programs.

## Materials and Methods

### *Phytophthora* Infection Assays

*Solanum tuberosum* differential host ‘Ma*R3*,’ a susceptible potato line, was used to prepare the infection materials. Potato seedlings were cultured in sterile Murashige and Skoog medium for a month, then transferred to vermiculite for another month, before being planted in pots containing a mix of peat moss and vermiculite (V/V = 2:1). After 5 weeks, the fully expanded leaves were used for inoculation. *P*. *infestans* strains were cultured and maintained on RSA (rye sucrose agar) medium plates. They were grown at 16°C in darkness for 2 weeks on RSA plates, then sporangia were harvested in cold sterile distilled water. The sporangial suspensions were adjusted to 4 × 10^4^ spores mL^-1^ with distilled water and chilled at 4°C for 2 h to release motile zoospores (Tian et al., 2015a). The detached potato leaves were inoculated with the zoospore suspension, placed in a plastic tray, and maintained at 16°C and 100% relative humidity in the darkness to ensure infection. The infected leaves were observed under a microscope, pooled at 12 h post inoculation (hpi), frozen with liquid nitrogen, and stored at -80°C.

### Trypan Blue Staining

Leaf inoculation sites were cut off and transferred into polypropylene tubes which were filled with diluted trypan blue solution (10 g phenol, 10 mL glycerol, 10 mL lactic acid, 10 mL water and 10 mg of trypan blue). The tubes (lid slightly unscrewed) were treated in a heated water bath and boiled for 2 min. After cooling to room temperature, tubes were boiled for another 2 min. Then, the samples were destained by replacing the staining solution with chloral hydrate solution (5 g chloral hydrate dissolving into 2 mL water) for 24 h. The samples were finally mounted in distilled water and viewed under an Olympus BX51 (Shinjuku-ku, Tokyo, Japan) microscope with differential interference contrast optics (Meng et al., 2015).

### RNA Isolation and Sequencing

Total RNA was extracted from the infection tissues using TRIzol reagent (Invitrogen, Carlsbad, CA, United States) following the manufacturer’s protocol, then treated with DNase I (RNase free, TaKaRa, Japan) to remove genomic DNA contaminations. The quality, purity and concentration of each RNA sample were checked by using the 1% agarose gels, NanoDrop 2000c spectrophotometer (Thermo Scientific, Waltham, MA, United States) and Agilent 2100 bioanalyzer (Agilent Technologies, Santa Clara, CA, United States). Library preparation was performed from 1 μg of total RNA. Sequencing were conducted on Illumina HiSeq 2000 platform to produce100 bp paired-end reads (LC Company, Hangzhou, China). The sequencing results were deposited in the Sequence Read Archive (SRA) at the NCBI database (accession number: PRJNA415282).

### RNA-Seq Data Analysis

The adapter sequence and low quality bases were trimmed from both ends of reads with the software HTQC (Yang et al., 2013). The command ‘ht-stat’ was used to assess the quality of reads. The command ‘ht-trim -S both -C 13 -W 5’ was used to trim both sides of reads. Then, the command ‘ht-filter –filter length -L 35’ was used to filter out short reads. The *P*. *infestans* reference genome of strain T30-4 was downloaded from the PID (*Phytophthora infestans* Database)^1^. TopHat2 was used to map the clean reads to the reference genome with the custom parameter setting ‘-r 0 –mate-std-dev 80’ (Trapnell et al., 2012). Cufflink was used to calculate the Fragments Per Kilobase of exon model per Million mapped reads (FPKM) of each sample. SAMtools was used to extract SNPs from the accepted.bam file, with parameters set as ‘DP = 4, QUAL = 20’, with other parameters set as default. MEGA6.0 was used to perform the sequences alignment analysis and construct the phylogenetic tree (Tamura et al., 2013).

### Prediction of Novel RXLR Effector Genes

Pair-end reads were mapped to the genome of *S*. *tuberosum* using TopHat2 (Kim et al., 2013). Then, reads that could not be mapped to the potato genome were extracted and we performed *de novo* assembly with Trinity (Grabherr et al., 2011). The longest potential coding regions were identified by TransDecoder (Haas et al., 2013), then the sequence was trimmed before the first methionine. Sequences more than 70 bp long were analyzed with a python script to predict the RXLR proteins following the reported rules (Whisson et al., 2007).

### qRT-PCR

For RNA-seq data validation, SYBR green qRT-PCR assays were performed. Primer pairs (Supplementary Table 1) were designed to anneal specifically to each of the selected genes. The housekeeping gene of *P*. *infestans*, *Pi*UBC (PITG_08327), was used as endogenous control. Correlation analysis of RXLR genes were conducted by associating the ΔCt values calculated from the qRT-PCR assays with the log_2_-transformed expression levels from the RNA-seq data.

### Transient Agro-Infiltration Assays

RXLR effector genes and *GFP* gene were amplified with primers containing *Cla*I and *Sal*I restriction sites and ligated into PVX vector pGR106 (with 35S promoter) using standard molecular biology techniques. For *in planta* transient gene expression assays, *A*. *tumefaciens* strain GV3101, with the appropriate constructs, were grown in LB media to the late-log phase (supplemented with 50 μg^*cdot*^mL^-1^ of kanamycin and 20 μg^*cdot*^mL^-1^ of rifampicin). The cells were collected by centrifugation at room temperature (25°C, 3500 g, 5 min), resuspended in an infiltration medium (200 μM acetosyringone, 10 mM MES, pH 5.6, and 10 mM MgCl_2_), and then incubated for 1–3 h at room temperature before infiltration. *A*. *tumefaciens* suspensions were infiltrated at an OD_600_ value of 0.3–0.6. Agroinfiltration experiments were performed on leaves of 6- to 8-week-old *N*. *benthamiana* plants and 5-week-old *S*. *tuberosum* plants. After 24 h, infiltrated *N*. *benthamiana* leaves were detached and transferred into moist sealed plastic trays and inoculated with 10 μl *P*. *infestans* zoospore suspension (100 zoospores/μl) at the infiltration sites (Tian et al., 2016). *P*. *infestans* strain Pa21106 was used in the infection assay. Lesion diameters of the inoculated leaves were recorded and photographs were taken 5 days after infiltration (Gu et al., 2011).

To assay suppression of HR, *A*. *tumefaciens* cells carrying selected RXLR effector genes were infiltrated. About 24 h later, the same infiltration site was infiltrated with *A*. *tumefaciens* cells carrying the *Bax* gene. *A*. *tumefaciens* cells carrying *Bax*, or RXLR effector or *GFP* genes alone, were infiltrated in parallel as controls. Plants were grown and maintained in a cultivation room with an ambient temperature of 22–25°C and high light intensity throughout the experiments. Symptom development was observed from day 3 to day 8. Statistics were performed and photographs taken at day 5. Similar procedures were used for other cell death suppression assays, where, *A*. *tumefaciens* cells carrying *Bax*, *NIP*, *Avh238*, *Avh241*, or *INF1* were infiltrated 24 h after the RXLR effector constructs were infiltrated (Wang et al., 2011). Each assay consisted of three biological replications (Gu et al., 2011).

## Results

### Five Diverse Strains Were Selected for Analysis of RXLR Effector Genes

**FIGURE 1:**
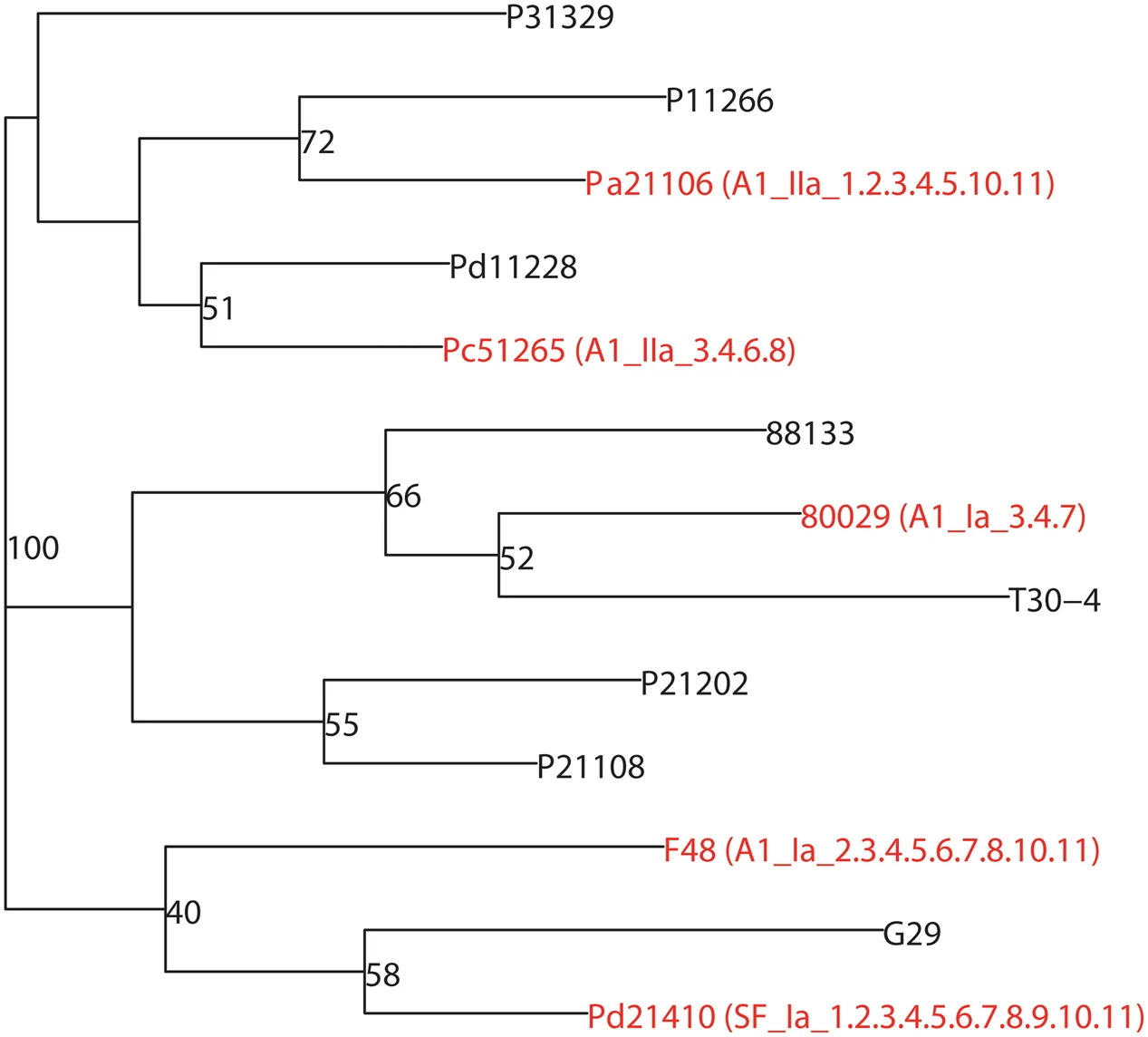
Five diverse *P*. *infestans* strains with highly divergent genetic backgrounds were chosen for RNA-seq. Thirteen representative strains were used to build a phylogenetic tree according to the Bruvo distance using the R package “poppor” (Bruvo et al., 2004). The strains 80029, F48, Pa21106, Pc51265, and Pd21410 marked as red branches in the figure, were selected to conduct RNA-seq. Mating type: A1 and SF (self-fertilization); haplotype: Ia and IIa; pathotype: virulence race indicated by Black’s potato differentials.

In our previous studies, more than 2,000 *P*. *infestans* isolates were collected and a population analysis was conducted (Tian et al., 2015a,b, 2016). Based on SSR genotype data, a phylogenetic tree containing thirteen representative strains was constructed (Supplementary Table 2). As shown in **Figure 1**, five diverse strains Pa21106, Pc51265 and Pd21410 (collected from a north-western China population), F48 (collected from a southern China population) and 80029 (collected from European population and used as reference strain) with different mating types, haplotypes, and pathotypes, were selected and used in the following work.

### Leaves 12 hpi Were Chosen to Prepare Early *in Planta* Materials

**FIGURE 2:**
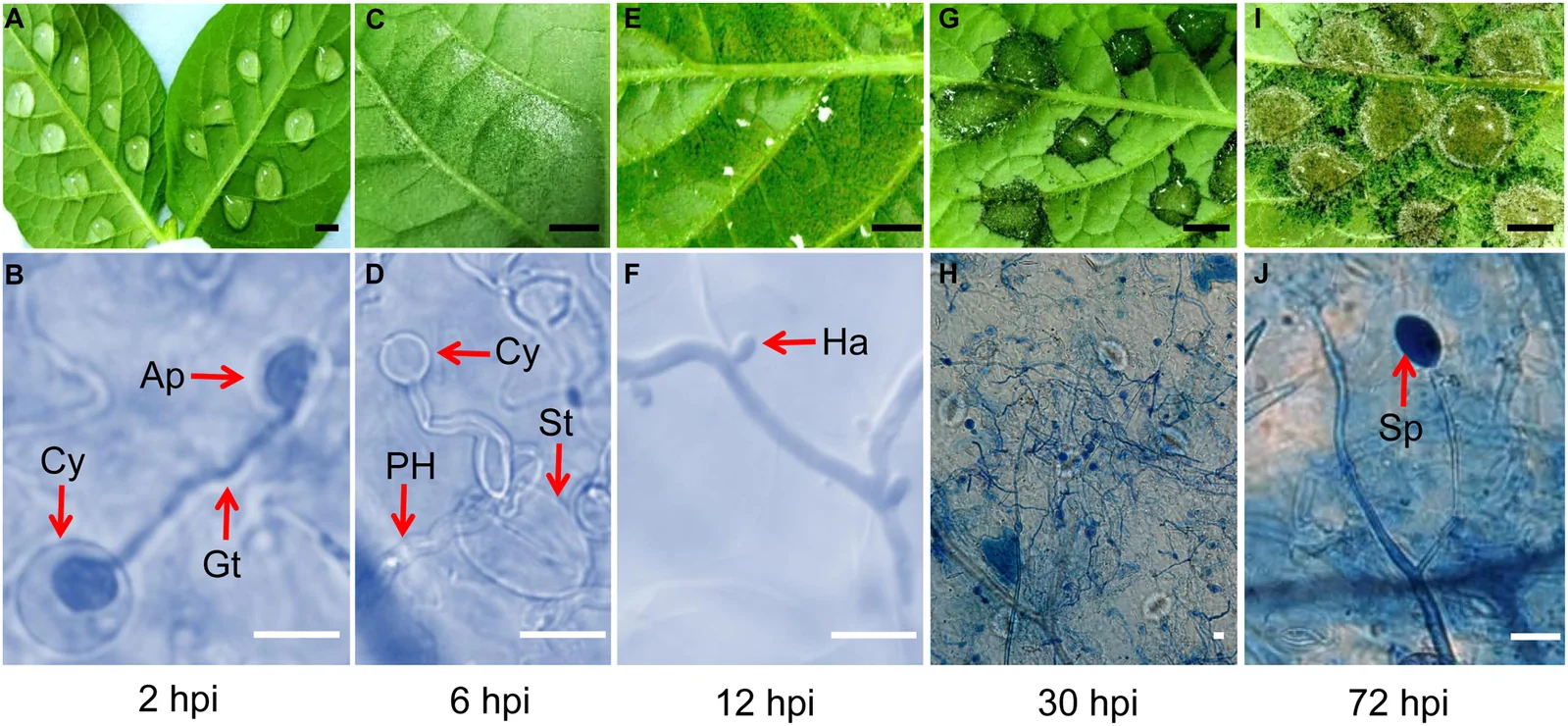
The ideal sampling time point of potato infection by *P. infestans* was confirmed by observation. **(A,B)** At 2 h post inoculation (hpi), the Cy (cyst) germinated and extended out Gt (germ tube) to form Ap (appressorium) and was ready to invade into host cells. **(C,D)** At 6 hpi, some pathogen PH (primary hyphae) invaded into host through the host St (stoma), and developed inside the host. **(E,F)** At 12 hpi, young hyphae broadly spread and haustoria formed, but only very small black spots could be macroscopically observed on the leaf. **(G,H)** At 30 hpi, the infection site turned to dark and obvious damage could be observed. **(I,J)** At 72 hpi, air hyphae appeared and young Sp (sporangium) was ready to form. (Black bar, 0.5 cm; white bar, 10 μm).

Inoculated potato leaf materials were continuously harvested at different time points. Macroscopic and microscopic observations were used to monitor the disease development process. At 2 hpi, the resting cysts of *P*. *infestans* germinated and extended geminating tubes. The appressoria were formed at the end of the tubes. No obvious leaf symptoms were observed (**Figures 2A,B**). At 6 hpi, the appressoria were observed to extend primary hypha and pass through the host stomata into the host tissues. Light water-soaked appearances were seen at the inoculation sites (**Figures 2C,D**). At 12 hpi, as in a previous report (Sivak and Shaw, 1969), haustoria were observed, which means that colonization had begun. Small dark spots were observed on leaves (**Figures 2E,F**). At 30 hpi, secondary mycelia were formed and widely expanded. The well-formed water-soaked lesions were observed at the infection sites (**Figures 2G,H**). At 72 hpi, aerial mycelia were observed surrounding the infection sites and young sporangia appeared in sporangiophores (**Figures 2I,J**). Based on these results, the time point of 12 hpi was chosen to prepare the early *in planta* materials to perform *P*. *infestans* deep sequencing.

### Detection of 245 RXLR Effector Genes Expressed at the Early Infection Stage

To understand genetic differences in RXLR effector genes, the five genetically diverse strains of *P*. *infestans* were used to prepare *in planta* infections. The inoculated leaf tissues were sampled at 12 hpi and total RNA was isolated for RNA sequencing. In total, 379 million raw pair-end reads were produced. After filtering, mapping, and gene assembling, 11,911 unique expressed genes were detected, including 243 RXLR effector genes (Supplementary Table 3). Recently, Cooke et al. (2012) and Martin et al. (2013) reported eleven additional RXLR genes. Therefore, sequencing reads were parallel-mapped to these eleven genes and one of them, *PiRXLRe*, was found to be expressed in Pa21106, as two unique reads of Pa21106 were successfully mapped to the gene. To confirm whether our strains had unique RXLR effector genes, RNA-seq reads were also used to carry out *de novo* assembly and RXLR effector gene prediction. One novel RXLR effector gene, comp858_c1_seq1 (GenBank accession number: MG269997), was identified in Pa21106 (Supplementary Table 4). In total, in this study, expression of 245 RXLR effector genes was detected in early infection stage *in planta*.

### Expression and Sequence Analysis Uncovered 18 Candidate Core RLXR Effectors

RNA-seq data were partially validated by qRT-PCR assays. The transcript abundances determined by qRT-PCR were highly consistent with RNA-seq results, indicating that the RNA-seq data were reliable (*R*^2^ = 0.8783, *p*-value < 0.01) (Supplementary Figure 1). Among the detected 245 RXLR effector genes, 108 were expressed in all five strains, 34 in four strains, 29 in three strains, 30 in two strains, and 44 in one strain (Supplementary Figure 2). Meanwhile, SNPs extracted from our RNA-seq reads and from *P*. *infestans* strain 06_3928A resequencing reads (Cooke et al., 2012) were used to analyze RXLR effector gene sequence polymorphisms. In total, 468 SNPs were detected in 128 RXLR effector genes, resulting in the discovery of 115 potentially conserved RXLR effector genes, including 92 with no SNPs and 23 with synonymous substitutions (Supplementary Table 3). Furthermore, 23 RXLR effector genes that were highly expressed and potentially conserved were extracted; they were both conserved in protein coding sequences and expressed by all five strains at the early infection stage. Although 23 RXLR effector genes have been assigned different gene IDs by Haas et al. (2009), subsequent phylogenetic analysis of these genes showed that the genes PITG_14954, PITG_14959, PITG_14961, and PITG_14962 (CRE11), PITG_16233, and PITG_16240 (CRE13), PITG_23014, and PITG_23015 (CRE16) have consensus sequences. Since effector genes with same sequences can be considered one core RXLR effector, only 18 core RXLR effectors were uncovered (**Table 2** and Supplementary Figure 3).

### Core RLXR Effectors Contribute to Virulence through Defense Suppression

**FIGURE 3:**
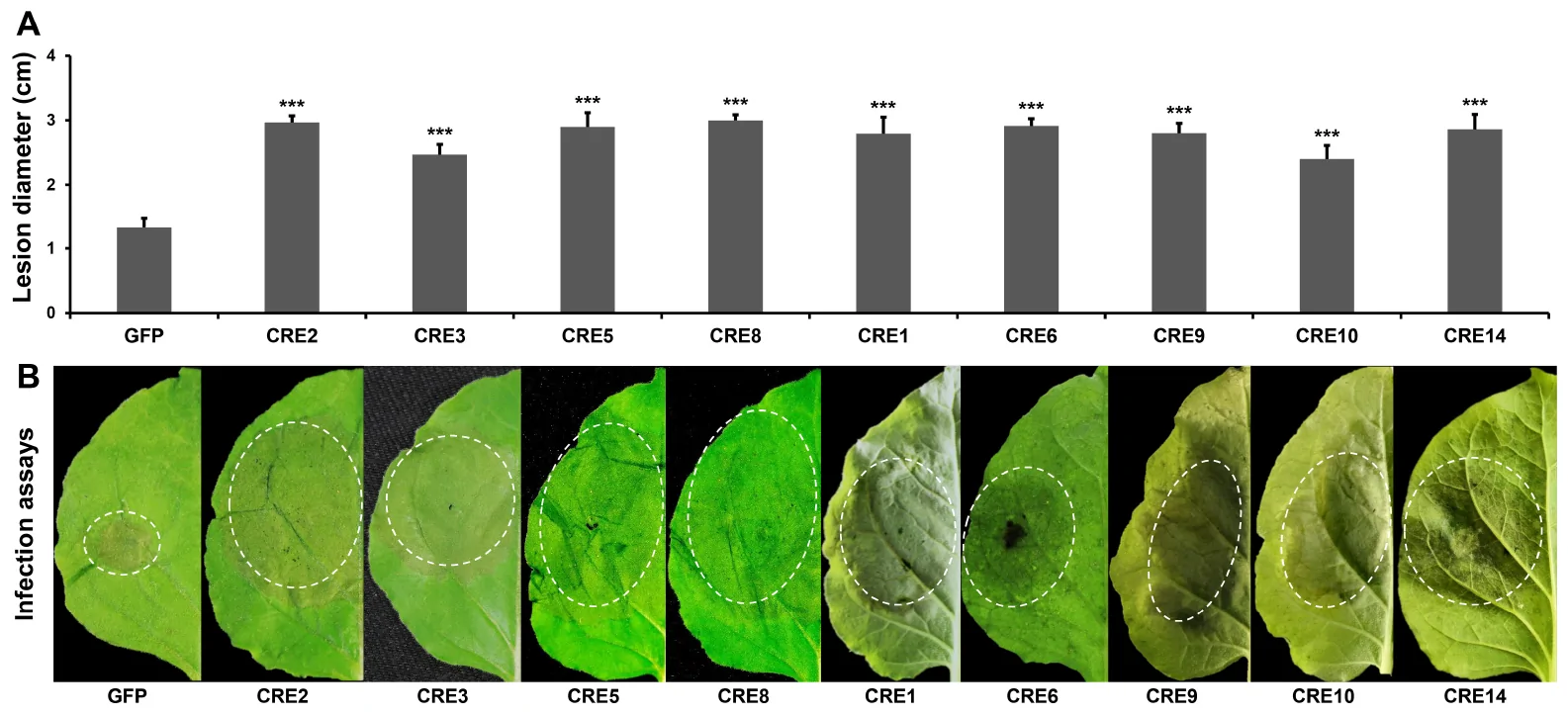
Transient overexpression of selected RXLR effector genes enhances *P*. *infestans* colonization. **(A)** Average lesion diameters of the inoculated leaves. Error bars represent standard errors calculated from at least 20 independent biological replicates. Asterisks indicate significant differences determined using Dunnett’s test (*P* < 0.005), **(B)** *N*. *benthamiana* leaf phenotypes upon *P*. *infestans* infection. Detached leaves of *GFP*- and RXLR effector-transgenic plants were inoculated with *P*. *infestans* zoospores. These representative photographs were taken at 120 hpi.

**FIGURE 4:**
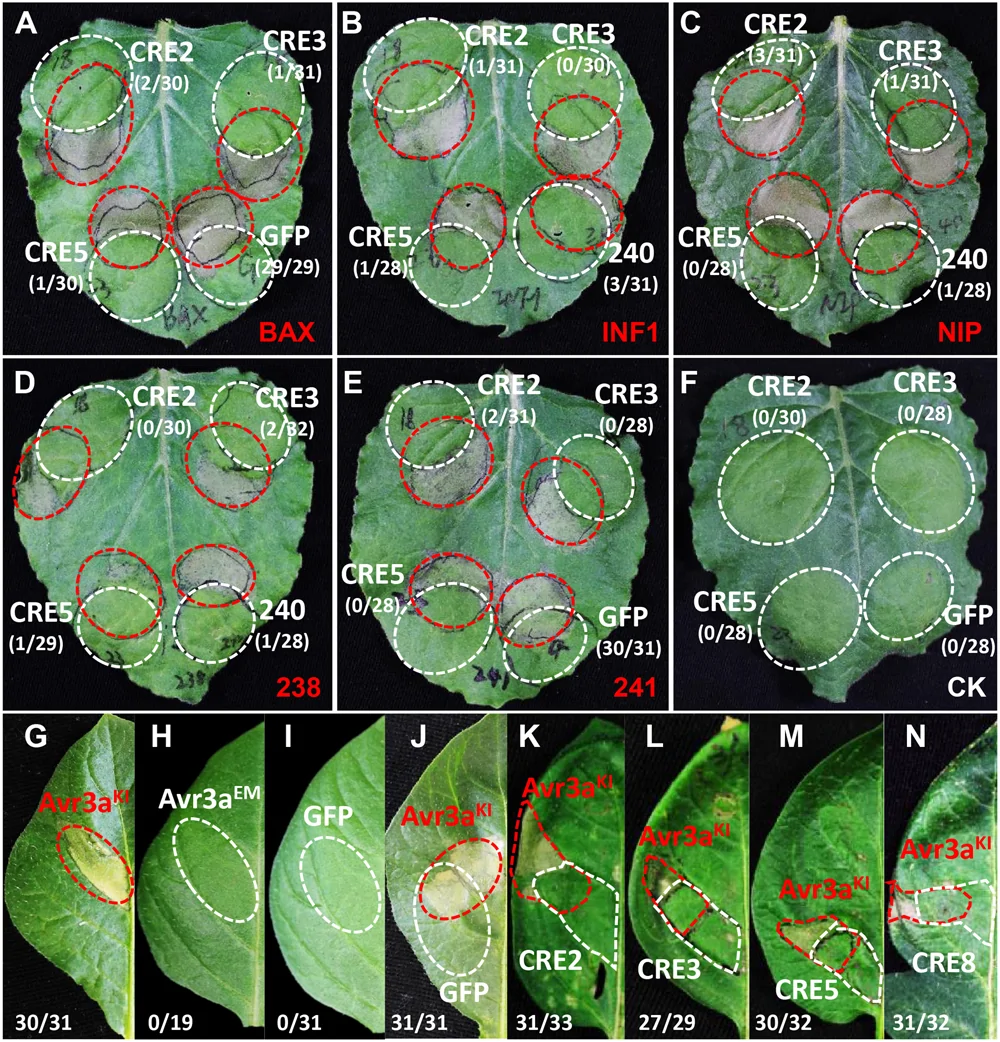
Plant defense suppression activities of selected *P*. *infestans* RXLR effector genes. Candidate RXLR effector genes that were highly expressed at the early stage of plant infection were selected and *A*. *tumefaciens*-mediated transient expression assays were performed on *N*. *benthamiana* **(A–F)** and host potato **(G–N)**. In *N*. *benthamiana*, all examined RXLR effector genes suppressed HR mediated by a range of elicitors including: **(A)** BAX; **(B)** the *P*. *infestans* PAMP elicitor INF1; **(C)** NIP; the *P*. *sojae* RXLR effectors Avh238 **(D,E)** Avh241. The red circle indicates the area infiltrated with *A*. *tumefaciens* cells carrying elicitor constructs. The white circle indicates *A*. *tumefaciens* cells carrying the RXLR effector gene *CRE2* (PITG_04196), *CRE3* (PITG_05750), and *CRE5* (PITG_06308), the negative control GFP **(A,E)**, and the positive control Avh240 **(B–D)**. In *S*. *tuberosum*, the *P*. *infestans* RXLR effector Avr3a^KI^ induces HR in the differential host line ‘Ma*R3*,’ which was suppressed by transient expression of RXLR effector genes **(K)** *CRE2*, **(L)** *CRE3*, **(M)** *CRE5*, and **(N)** *CRE8* (PITG_09160); **(G)** the positive control Avr3a^KI^, and **(H–J)** the negative control Avr3a^EM^, GFP, and Avr3a^KI^ plus GFP. The numbers show the ratio of infiltrated sites that developed cell death versus the total number of infiltrated sites.

To examine whether the core RXLR effectors promote *P*. *infestans* virulence, the mature protein-coding regions of PITG_04196, PITG_05750, PITG_06308, PITG_09160, PITG_00821, PITG_07451, PITG_09224, PITG_13452, and PITG_17063 were cloned and transiently expressed in *N*. *benthamiana* using *Agrobacterium*-mediated expression followed by a *P*. *infestans* challenge. At 5 days post inoculation (dpi), significantly larger lesions were observed in areas expressing RXLR effectors compared to that of free GFP (**Figure 3**), suggesting that the core RLXR effectors confer a benefit to the pathogen. To examine how the core RXLR effectors promote virulence, constructs of PITG_04196, PITG_05750, PITG_06308 and PITG_09160 were randomly selected to test cell death suppressive activities triggered by PAMPs and effectors, using their *Agrobacterium*-mediated transient expression in leaves of *N*. *benthamiana*. None of the four genes induced cell death in *N*. *benthamiana*. In fact, comparing to negative control PITG_22804 and free GFP, they all suppressed the cell death caused by a range of cell death inducers, including BAX, INF1, NIP, Avh238 and Avh241 (Wang et al., 2011). These results indicated that these conserved core RXLR effectors contribute to virulence during the early infection stage (12 hpi), by inhibiting plant defense responses induced by both PTI and ETI (**Figures 4A–F** and Supplementary Figure 3). The functional characterization of these four RXLR effector genes was also conducted on leaves of potato cultivar ‘Ma*R3*,’ *S*. *tuberosum*. Comparing to negative control Avr3a^EM^ and free GFP, none of them induced cell death, but could suppress HR caused by transient expression of Avr3a^KI^, suggesting that these conserved core RXLR effectors that are expressed at the early infection stage are important in the suppression of ETI-induced host plant defense (**Figures 4G–N**).

### Analysis of 10 Known *Avr* and 8 *SFI* RXLR Effector Genes

Previously, 10 known *Avr* RXLR effector genes had been reported (Vleeshouwers and Oliver, 2014). In this study, their expression at early stage of potato infection was detected. *Avr1* was only found to be expressed in strain 80029. *Avr2*, *Avr3a*, *Avrblb1*, *Avrvnt1*, and *AvrSmira1* were all expressed in all five tested *P*. *infestans* strains, while *Avr3b*, *Avr4*, *Avrblb2* and *AvrSmira2* were each expressed in two to four strains. Sequence polymorphism analyses showed that the *Avr3a*^EM^ allele and the truncated *Avr4* were expressed by the tested strains (**Table 3** and Supplementary Figure 5).

Recently, Zheng et al. (2014) reported 8 *SFI* (Suppressor of early Flg22-induced Immune response) RXLR effector genes. Here, their expression was also detected, with *SFI2*, *SFI3*, and *SFI4* being highly expressed in all five strains. *SFI5* was expressed at a low level in four strains, *SFI1* and *SFI6* were not expressed in any strains, and *SFI8* was only expressed at a low level in strain Pa21106. The expression of *SFI7* was very distinct among individual strains, either highly expressed or completely undetectable in any given strains (Supplementary Figure 5).

## Discussion

Core effectors were considered ideal target for deploying durable resistance, but *P*. *infestans* core RXLR effectors are largely unknown. In this study, to lay the foundation for searching for durable potato *R* genes and long-lasting controlling late blight disease, we utilized next-generation transcriptome deep sequencing strategy to identify potentially conserved core RXLR effector genes in *P*. *infestans*, primarily based on their (1) conserved sequences among diverse strains in the population, (2) high levels of expression in the early infection stage (12 hpi), and (3) potentially essential functions in pathogenesis.

In our previous studies, more than 2,000 *P*. *infestans* strains were collected from a broad potato cultivation area of China from 2008 to 2013 (Tian et al., 2015a,b, 2016). Their population structures were analyzed, which helped us to further identify core RXLR effectors at the population level. According to previous population analysis data (e.g., SSR genotype, mtDNA haplotype, mating type and pathotype), four representative strains Pa21106, Pc51265, Pd21410 (collected from northwestern China population), and F48 (collected from southern China population) were selected for further analysis. Meanwhile, since *P*. *infestans* populations in northwestern China were genetically distant from European lineages (Tian et al., 2016), a representative strain, named 80029, from European population was also used in our study. *P*. *infestans* RXLR effectors have been reported to be extremely up-regulated during infection and colonization (Cooke et al., 2012). Thus, in this study *in planta* infection stage materials of five diverse *P*. *infestans* strains were used to conduct transcriptome deep sequencing (**Figure 1** and Supplementary Table 2). In addition, 12 hpi *in*-*planta* materials were collected for RNA-seq, because this is the time point at which the haustorium started to form (**Figure 2**), which means that colonization was established and the delivery of effectors into plant cells was underway (Sivak and Shaw, 1969; Petre and Kamoun, 2014).

In this study, deep-sequencing approach produced over four hundred million reads from five samples. Among them, more than 2 million reads were successfully mapped to the *P*. *infestans* reference genome, and 245 expressed RXLR effectors genes were detected (**Table 1** and Supplementary Table 3), much more than that in previous studies in tomato-*P. infestans* interaction materials (79 effector genes) and potato-*P. infestans* interaction materials (31 effector genes) (Haas et al., 2009; Zuluaga et al., 2015). One reason for these results is the difference between the microarray and Illumina sequencing platforms (Haas et al., 2009). Another reason is the difference in the quantity of data between our sequencing and others (Zuluaga et al., 2015).

**Table 1:** Deep sequencing data.

Strain	Raw	Clean	Mapped	Ratio	No. of
	reads	reads	reads	(%)^a^	RXLR^b^
80029	81,170,140	78,088,447	209,151	0.268	166
F48	76,156,500	67,813,237	121,189	0.179	147
Pa21106	71,809,644	62,860,454	1,577,134	2.509	226
Pc51265	60,289,336	53,667,578	189,841	0.354	167
Pd21410	89,624,368	79,813,868	147,278	0.185	162
All	379,049,988	342,243,584	2,244,593	0.656	245

^a^ *The percentage of clean reads belonging to *P. infestans* in certain sample.*

^b^*Number of RXLR effector genes detected in certain strain*.

Expression of the 245 RXLR effector genes was comparably at high levels, consistent with the fact that RXLR effectors are *in planta* induced genes and are up-regulated during infection and colonization (Cooke et al., 2012) (Supplementary Figure 2A). Interestingly, among these expressed RXLR effector genes, most (108) were expressed by all strains and a large number (44) were specifically expressed by just one strain (Supplementary Figure 2B). Cooke et al. (2012) compared the RXLR expression profiles and aggressiveness of three *P. infestans* strains and found that highly aggressive strains expressed more RXLR effector genes. They, therefore, speculated that RXLR were likely virulence determinants that enhance aggressivenesses. Accordingly, we speculate that, as virulence factors, the expressed RXLR effectors may have partial contributions to fundamental pathogenesis and the uniquely expressed RXLR effectors may have partial contributions to divergence in virulence. However, additional work is required to determine exactly which genes contribute virulence to any particular strain.

In addition, to obtain conserved core RXLR effectors from *P*. *infestans*, 47 of the 108 RXLR effector genes with higher expression levels were selected for subsequent analysis. Bart et al. (2012) conducted SNP analysis to select conserved core effectors. Similarly, in this study, the sequence polymorphism analysis was performed by comparing SNPs and dN/dS ratios of 245 RXLR effector genes among strains. This led to the identification of 23 RXLR effector genes that are highly expressed at the early infection stage and conserved in protein coding sequences. Phylogenetic analysis of these 23 genes revealed that RXLR effector genes, PITG_14954, PITG_14959, PITG_14961, and PITG_14962 (CRE11), PITG_16233, and PITG_16240 (CRE13), PITG_23014, and PITG_23015 (CRE16), were designated different gene IDs yet shared consensus nucleotide sequences (Haas et al., 2009). Thus, these 23 genes were categorized as 18 conserved core RXLR effectors (**Table 2** and Supplementary Figure 2).

**Table 2:** Identification of 18 conserved candidate core RXLR effector genes.

Name^a^	Gene ID^b^	Family^c^	Intergenic^i^	Five strains^d^				06_3928A^e^			
				Snp	N^f^	S^g^	dN/dS^h^	Snp	N	S	dN/dS
CRE1^h^	PITG_00821	RxLRfam108	InBtw	0	0	0	NA	0	0	0	NA
CRE2	PITG_04196	RxLRfam47	GSR	0	0	0	NA	0	0	0	NA
CRE3	PITG_05750	RxLRfam29	InBtw	0	0	0	NA	0	0	0	NA
CRE4	PITG_05910	RxLRfam52	InBtw	0	0	0	NA	0	0	0	NA
CRE5	PITG_06308	RxLRfam23	InBtw	0	0	0	NA	0	0	0	N/A
CRE6	PITG_07451	RxLRfam116	InBtw	1	0	1	–1	0	0	0	NA
CRE7	PITG_08133	RxLRsng158	InBtw	0	0	0	NA	0	0	0	NA
CRE8	PITG_09160	RxLRfam42	GSR	0	0	0	NA	0	0	0	N/A
CRE9	PITG_09224	RxLRfam55	InBtw	1	0	1	–3.9521	0	0	0	N/A
CRE10	PITG_13452	RxLRfam108	InBtw	0	0	0	NA	0	0	0	NA
CRE11	PITG_14954	RxLRfam21	GSR	0	0	0	NA	0	0	0	N/A
	PITG_14959	RxLRfam21	GSR	0	0	0	NA	0	0	0	N/A
	PITG_14961	RxLRfam21	GSR	0	0	0	NA	0	0	0	N/A
	PITG_14962	RxLRfam21	GSR	0	0	0	NA	0	0	0	N/A
CRE12	PITG_14960	RxLRfam21	GSR	0	0	0	NA	0	0	0	NA
CRE13	PITG_16233	RxLRfam9	InBtw	0	0	0	NA	0	0	0	NA
	PITG_16240	RxLRfam9	InBtw	0	0	0	NA	0	0	0	N/A
CRE14	PITG_17063	RxLRfam45	Not	0	0	0	NA	0	0	0	NA
CRE15	PITG_17316	RxLRfam1	InBtw	1	0	1	–2.6297	1	0	1	0
CRE16	PITG_23014	RxLRfam100	GSR	0	0	0	NA	0	0	0	N/A
	PITG_23015	RxLRfam100	GSR	0	0	0	NA	0	0	0	NA
CRE17	PITG_23042	RxLRfam25	GDR	0	0	0	NA	0	0	0	N/A
CRE18	PITG_23226	RxLRfam100	Not	0	0	0	NA	0	0	0	NA

^a,h^*Designated names of core RXLR effector (CRE) genes.* ^b^*Gene ID of candidate conserved RXLR effectors in reference genome T30-4 (Haas et al., 2009).* ^c^*RXLR effector gene families.* ^d^*The five diverse *P*. *infestans* strains used in our study (Pa21106, Pc51265, Pd21410, F48 and 80029).* ^e^*P. *infestans* strains used in the study of Cooke et al. (2012).* ^f^*Non-synonymous substitution.* ^g^*Synonymous substitution.* ^h^*The ratio of non-synonymous substitution rate to synonymous substitution rate.* ^i^*GSR (gene spare region), GDR (gene density region), and InBTw (gene in the region between GSR and GDR) are three types of intergenic regions*.

Core effectors should make substantial contributions to pathogen virulence (Dangl et al., 2013). To validate the virulence contribution of the core RXLR effectors uncovered in this study, nine of the 18 candidate core effectors were selected and transiently expressed in *N*. *benthamiana*, then challenged with *P. infestans*. Results revealed that areas infiltrated with effectors showed significantly larger lesion comparing to free GFP control, which showed that those core effectors contribute to virulence during plant-pathogen interaction (**Figure 3**). BAX, INF1, NIP, Avh238 and Avh241 are all of cell death inducers that can activate plant PTI and ETI defenses and widely used in examining effector virulence functions (Wang et al., 2011). To further explore these virulence functions, the candidate core effectors were transiently expressed in *N*. *benthamiana*, then challenged by infiltrating cell death inducers at the same areas. Results showed that the core effectors could suppress the cell death caused by a range of inducers, suggesting that all four examined RXLR effector genes suppressed plant defenses mediated by both PTI and ETI (**Figures 4A–F** and Supplementary Figure 4). Meanwhile, cell death suppression assays were also tested on host potatoes. Potato differential host ‘Ma*R3*’ contains resistance gene *R3*, which can recognize *Avr3a^KI^* to activate host defense response that results in cell death. Cell death suppression assays in ‘Ma*R3*’ showed that the examined RXLR effector genes could suppress the HR caused by transient expression of *Avr3a^KI^*, suggesting that these conserved core RXLR effectors expressed at early infection stages are important in the suppression of host plant defenses induced by ETI (**Figures 4G–N** and Supplementary Figure 4).

Cooke et al. (2012) previously identified 45 core RXLR effectors showing *in planta* gene induction at 2 or 3 dpi in all three examined strains (06_3924A, NL07434, and T30-4), including 5 *Avr* genes with known gain-of-virulence variants (Vleeshouwers and Oliver, 2014). In this study, a collection of 18 core RXLR effectors, which are highly expressed at the early infection stage (12 hpi) and have sequences that are conserved among diverse strains, was identified. No previously known *Avr* RXLR effector genes were among these 18 core effectors. Meanwhile, transient expression assays in *N*. *benthamiana* and *S*. *tuberosum* showed that these tested candidate core RXLR effector genes have potentially important contributions to virulence (**Figures 3**, **4**). These results are consistent with the fact that genes with important functions are evolutionary conserved and under negative selection (Dangl et al., 2013). More importantly, the 18 core effectors identified in this study are highly expressed at 12 hpi, suggesting that they could activate host defenses at the early infection stage, when plants contain cognate *R* genes, through which disease development can be quickly constrained and potential disease-related losses can be decreased.

In *P. infestans*, 10 *Avr* RXLR effector genes had been reported and their gain-of-virulence alleles were analyzed (Vleeshouwers and Oliver, 2014). However, less information is available about their polymorphisms in Chinese *P*. *infestans* populations. Thus, in the present study, sequence polymorphisms and detected expression of these 10 A*vr* effector genes were performed in five high divergence stains to reveal their gain-of-virulence alleles (**Table 3** and Supplementary Figure 5). *Avr1* was only detected in strain 80029. Previously, Cooke et al. (2012) found that *Avr1* was not expressed in strain 06_3928A during the parasitic stage, because it was abandoned by the strain. Thus, we speculated that *Avr1* might also be lost by Chinese *P*. *infestans* strains, or that it does not express in the early infection stage. *Avr2* was highly expressed in all five examined strains. Sequence analyses showed an amino acid change (Asparagine to Lysine) at the 31st residue in strains 80029, F48, Pa21106, and Pc51265, but not in strain Pd21410. However, this change was reportedly not relevant to its *Avr* function (Gilroy et al., 2011). *Avr3a* was highly expressed in all five strains, and only the virulent form of E^80^M^103^ allele (80th residue Glutamic acid, 103rd residue Methionine) (Armstrong et al., 2005) was detected. *Avr4* was expressed at a low level in strains Pa21106 and F48, and a stop codon was found at 196 nt. Although the *Avr4* stop codon in strain 80029 was not detected in the present study due to its low level or complete lack of expression, it already has previously been found in this strain (van Poppel et al., 2008). Thus, the virulent truncated form of AVR4 is probably widely present in *P*. *infestans* populations. *Avrblb1* was highly expressed in all five examined strains. However, *ipiO4*, the suppressor of *Avrblb1*, was also highly expressed in these strains; it functions in suppressing the defense response mediated by *R* gene *Rpi*-*blb1* (Champouret et al., 2009; Chen et al., 2012). *Avrvnt1*, *AvrSmira1*, and *AvrSmira2* were highly expressed in all tested strains. SNPs were detected in these *Avr* genes, but, according to previous reports, the substitutions did not influence their *Avr* function (Pel, 2010; Rietman et al., 2012). In summary, Chinese *P*. *infestans* populations may still contain functional *Avr* effector genes that could be recognized by the matching *R* genes *R2*, *Rpi*-*blb2*, *Rpi*-*vnt1*, *Rpi*-*Smira1*, and *Rpi*-*Smira2*.

**Table 3:** Amino acid substitutions in the known RXLR effectors (AVR).

Amino acid position	Strains					
	T30-4	80029	F48	Pa21106	Pc51265	Pd21410
AVR2 (PITG_22870)						
31	N	K	K	K	K	N
AVR3a (PITG_14371)						
80	E	E	E	E	E	E
103	M	M	M	M	M	M
124	R	G	R	R	R	G
AVR4 (PITG_07387)						
19	T	-^a^	^∗b^	^∗^	-	-
AVRblb1 (PITG_21388)						
122	R	I	R	R	R	R
AVRblb2 (PITG_20300)						
79	P	-	-	P	A	-
AVRvnt1 (PITG_16294)						
96	A	A	V	A	-	A
AVRSmira1 (PITG_07550)						
131	K	K	K	R	R	R
170	R	R	R	Q	Q	R
199	K	T	K	T	K	T
209	H	H	H	R	H	H
AVRSmira2 (PITG_07555)						
64	M	V	–	M	M	M

^a^*Regions not covered by sequencing.* ^b^*Nucleotide sequence encodes a stop codon in this amino acid position*.

The expression of 8 Suppressor of early Flg22-induced Immune response (SFI) RXLR effectors, which have been reported to function in suppressing host immune response (Zheng et al., 2014), were also examined (Supplementary Figure 5). Results revealed that *SFI2*, *SFI3*, and *SFI4* were highly expressed in all five examined strains, suggesting their conserved function in interfering with plant immunity during the early infection stages. *SF1*, *SF6*, and *SFI8* were not expressed, or expressed at a low level, in examined strains, indicating that they do not function in the early infection stage. *SFI5* and *SFI7* may have divergent functions in different strains, as their expression was different among strains. For example, *SFI7* was highly expressed in strains 80029 and F48, but not expressed in strains Pa21106, Pc51265, or Pd21410. Although these 8 *SFIs* play virulent roles in suppressing PTI, they do not meet our standards for selecting core effectors (e.g., conserved protein coding sequences and/or being highly expressed at the early infection stage), suggesting that their virulence function may not be essential to *P*. *infestans* pathogenesis, at least not in the early infection stage.

The variation analysis of 10 avirulence and 8 virulence RXLR effectors suggests the possibility to monitor the pathogen virulence structure in population level and to trace down the virulence change based on the whole group of RXLR effector genes, which will help proper deployment of the resistance genes in the field to effectively control particular *P*. *infestans* population with cognate avirulence effectors. However, it should be noticed that, in our study, only one time point was used to profile the expression level of RXLR effector genes, while a time series sampling at early infection stage, such as 9, 12, and 15 hpi, would provide more information about the expression of RXLR effectors. In the future, we will expand the RNA-seq sequencing to more *P*. *infestans* strains and more sampling time points to reveal the expression pattern of RXLR effectors.

## Conclusion

In this research, five genetically diverse *P*. *infestans* strains were selected to identify core *P*. *infestans* RXLR effectors, which resulted in the discovery of 18 RXLR effectors that are conserved in sequence and highly and widely expressed in the early stage of plant infection. Functional characterization by transient gene expression confirmed that these core effectors have critical functions in suppressing plant defenses mediated by PTI and ETI. These candidate core RXLR effectors are valuable in searching for cognate *R* genes for durable resistance breeding, and for function and virulence target studies. RXLR effectors are emerging as tools to accelerate and improve the identification, functional characterization, and deployment of resistance genes for modern potato breeding (Vleeshouwers and Oliver, 2014). Our results will speed up the usage of core RXLR effectors in potato resistance breeding.

## Author Contributions

Conceived and designed the experiments: WS. Performed the experiments: JY, BG, GH, and YT. Analyzed the data: WS, JY, and HL-K. Contributed reagents/materials/analysis tools: JY, BG, GH, and JQ. Wrote the paper: WS and JY, with contributed from all authors.

## Conflict of Interest Statement

The authors declare that the research was conducted in the absence of any commercial or financial relationships that could be construed as a potential conflict of interest.

## Footnotes

1
http://www.broadinstitute.org/annotation/genome/phytophthora_infestans/Downloads.html
