# Correction: Conserved RXLR effector genes of *Phytophthora infestans* expressed at the early stage of potato infection are suppressive to host defense

**DOI:** 10.3389/fpls.2026.2012756

**Published:** 2026-10-13

**Authors:** Junliang Yin, Biao Gu, Guiyan Huang, Yuee Tian, Junli Quan, Hannele Lindqvist-Kreuze, Weixing Shan

**Affiliations:** 1 College of Plant Protection, Northwest A&F University, Xianyang, China; 2 State Key Laboratory of Crop Stress Biology for Arid Areas, Northwest A&F University, Xianyang, China; 3 College of Life Sciences, Northwest A&F University, Xianyang, China; 4 International Potato Center, Lima, Peru

**Keywords:** potato late blight, *Phytophthora infestans*, RXLR effectors, sequence polymorphism, durable resistance, resistance breeding

There was a mistake in **Figure 3** as published. SD/n rather than SD/√n was formerly used to calculate the SE, and the right SE values have been replaced in corrected **Figure 3A**. Besides, while preparing the composite **Figure 3B**, three images origin from same leaf were inadvertently placed in the affected panels of the CRE1, CRE6, and CRE9 sections to illustrate the disease lesions upon *Phytophthora infestans* infection. In the corrected **Figure 3B**, the CRE1 remains unchanged, and the affected CRE6 and CRE9 panels have been replaced with the appropriate available original image corresponding to the correct leaves. The corrected **Figure 3** appears below.

There was a mistake in the caption of **Figure 3** as published. While writing the caption, the independent biological replicates of “20” were wrongly typed as “30”. The corrected caption of **Figure 3** appears below.

**Figure 3:**
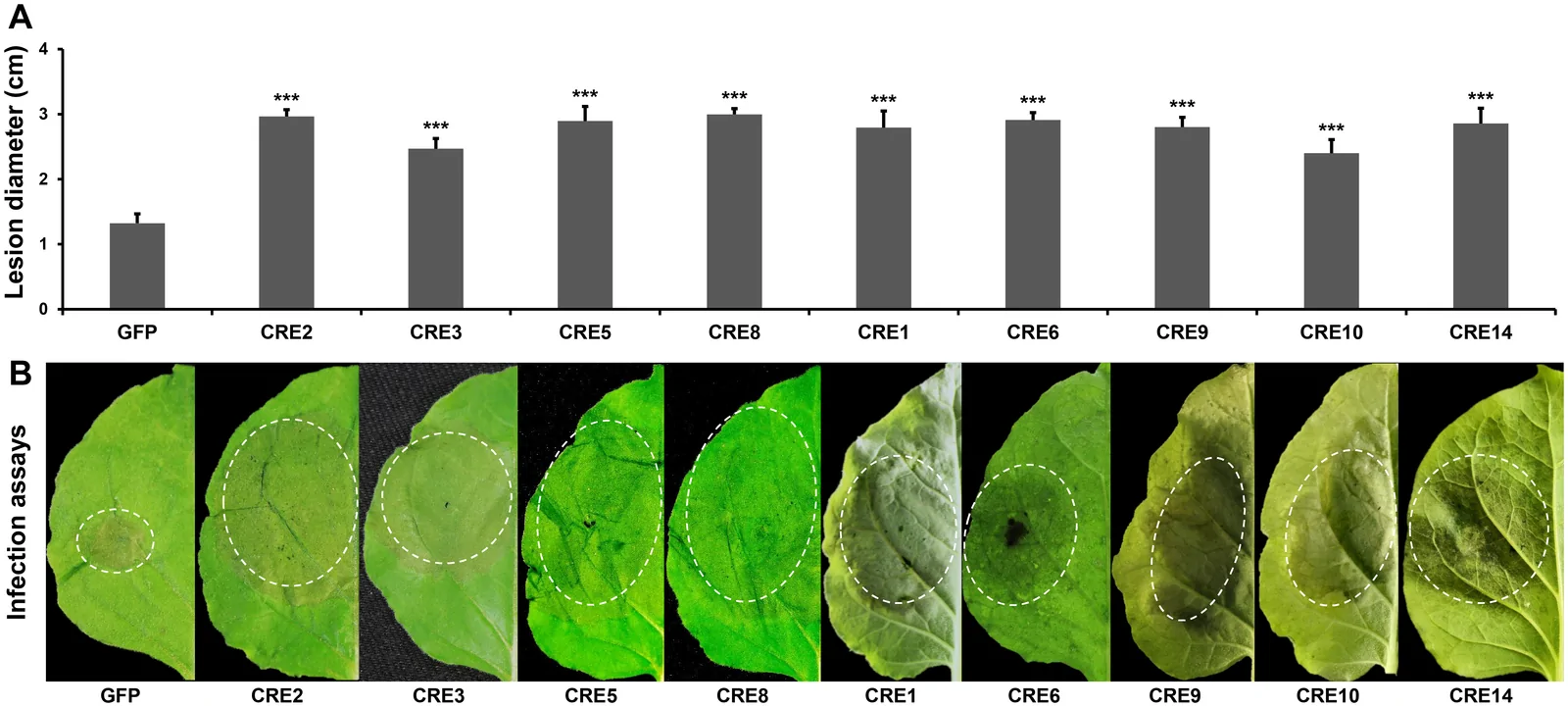
Transient overexpression of selected RXLR effector genes enhances *P. infestans* colonization. **(A)** Average lesion diameters of the inoculated leaves. Error bars represent standard errors calculated from at least 20 independent biological replicates. Asterisks indicate significant differences determined using Dunnett’s test (*P* < 0.005), **(B)** *N. benthamiana* leaf phenotypes upon *P. infestans* infection. Detached leaves of *GFP*- and RXLR effector-transgenic plants were inoculated with *P. infestans* zoospores. These representative photographs were taken at 120 hpi.

The original version of this article has been updated.

