# Supplementary material for: Conserved RXLR Effector Genes of Phytophthora infestans Expressed at the Early Stage of Potato Infection Are Suppressive to Host Defense
Source: Front Plant Sci. 2017 Dec 19;8:2155. doi: 10.3389/fpls.2017.02155 (PMC5742156; doi:10.3389/fpls.2017.02155)
Supplement: Supplementary file 1 [file Table_1.DOCX]

***Supplementary Material***

**Conserved RXLR effector genes of *Phytophthora infestans* expressed at the early stage of potato infection are suppressive to host defense**

**Conserved RXLR effector genes of *Phytophthora infestans* expressed at the early stage of potato infection are suppressive to host defense**

**Junliang Yin^1,3^, Biao Gu^1,3^, Guiyan Huang^2,3^, Yuee Tian^1,5^, Junli Quan^1,3^, Hannele Lindqvist-Kreuze^4^ and Weixing Shan^1,3^**

^1^College of Plant Protection, Northwest A&F University, Yangling, Shaanxi 712100, China

^2^College of Life Sciences, Northwest A&F University, Yangling, Shaanxi 712100, China

^3^State Key Laboratory of Crop Stress Biology for Arid Areas, Northwest A&F University, Yangling, Shaanxi 712100, China

^4^International Potato Center (CIP), Lima, Peru

^5^Present address: College of Forestry, Henan University of Science and Technology, Luoyang, Henan 471023, China

*** Correspondence:**Weixing Shan, College of Agronomy, Northwest A&F University, 3 Taicheng Road, Yangling, Shaanxi 712100, China
wxshan@nwafu.edu.cn

**Supplementary Tables**

**Supplementary Table 1. qRT-PCR primers for validating sequencing data.**

| Gene ID | Code | 5`-3` |
| --- | --- | --- |
| PITG_08327 | *Pi*UBCF | CATCAATCGGCGTATCTGTCTCA |
|  | *Pi*UBCR | CACCAAGTCGGCGAATAGCAC |
| PITG_09585 | *Pi*aF | ACAATGACCCCGTTATCCAA |
|  | *Pi*aR | CTTCTCTACCATCGCTACCG |
| PITG_09732 | *Pi*bF | TTTACTGCAAGCAACCTGAA |
|  | *Pi*bR | CACCAACCTGCGTAGACATC |
| PITG_18215 | *Pi*cF | GAGATTGGTTGATGCCCCG |
|  | *Pi*cR | ACATCTTTTCGCCCCTTACAG |
| PITG_22804 | *Pi*dF | AAATGTCAAAACTGGAACCC |
|  | *Pi*dR | AAACCTCTTAAATCCCGAAG |
| PITG_17063 | *Pi*eF | CACTCCAAGCAATCAAAGCG |
|  | *Pi*eR | GCCACTCACTGTAGAGCCTG |
| PITG_04196 | *Pi*fF | TGACTGAAGTGTCCGCATCC |
|  | *Pi*fR | GCTCTTCGCTTTCCGTGATT |
| PITG_14783 | *Pi*gF | GAGGTCTCTATCTAAAGCCGA |
|  | *Pi*gR | AAATGGTAGAAAGTAGGTGGC |
| PITG_07630 | *Pi*hF | AAAGAGGGGCTGCCGTTC |
|  | *Pi*hR | ATTGTGGGTTTCCTTGTATGTC |
| PITG_11507 | *Pi*iF | CGTTGCCGTCTCCCTTCTT |
|  | *Pi*iR | TCGTATGGGCTAGTTTCGC |
| PITG_09216 | *Pi*jF | TGAGACAGGCAATCACAGCA |
|  | *Pi*jR | ACCACCGATAGTAGCACCAA |

**Supplementary Table 2. Detailed information about SSR genotype, mating type, haplotype, and pathotype of representative strains.**

| Haplotype | Mating type | name | SSR primers and genotypes | | | | | | | | | | | | | | Race |
| --- | --- | --- | --- | --- | --- | --- | --- | --- | --- | --- | --- | --- | --- | --- | --- | --- | --- |
|  |  |  | Pi02 | Pi04 | Pi4B | Pi16 | Pi33 | Pi56 | Pi63 | Pi70 | Pi89 | D13 | G11 | SSR4 | SSR8 | SSR11 |  |
| Ia | A1 | T30-4 | 150/150 | 166/166 | 257/257 | 174/174 | 206/206 | 186/186 | 172/172 | 243/243 | 187/187 | 178/178 | 205/205 | 278/278 | 248/248 | 332/332 | ND |
| Ia | A1 | 80029 | 148/148 | 150/150 | 257/257 | 174/174 | 206/206 | 184/186 | 172/172 | 240/240 | 187/187 | 164/164 | 205/205 | 274/274 | 248/252 | 332/332 | 2.4.7 |
| Ia | A2 | 88133 | 150/150 | 152/158 | 255/255 | 174/174 | 206/206 | 184/184 | 170/170 | 240/240 | 187/187 | NA | 197/197 | 278/278 | 248/248 | 318/330 | 1.3.7.10.11 |
| IIa | A1 | Pa21106 | 146/146 | 158/158 | 251/259 | 174/174 | 209/209 | 184/184 | 172/172 | 240/240 | 187/187 | 164/164 | 199/199 | 270/270 | 256/256 | 330/342 | 1.2.3.4.5.6.7.8.9.10.11 |
| IIa | A1 | P11266 | 148/148 | 156/156 | 251/259 | 174/174 | 209/209 | 186/186 | 172/172 | 240/240 | 187/187 | 164/164 | 197/197 | 268/268 | 258/258 | 330/344 | ND |
| Ia | A2 | P21108 | 148/148 | 150/158 | 253/253 | 176/176 | 209/209 | 184/184 | 170/170 | 240/240 | 187/187 | 180/180 | 197/201 | 266/266 | 250/250 | 330/344 | ND |
| Ia | SF | P21202 | 150/150 | 158/158 | 253/253 | 174/174 | 206/206 | 184/184 | 170/170 | 237/237 | 187/187 | 180/180 | 197/201 | 266/266 | 250/250 | 334/344 | ND |
| IIa | A1 | P31329 | 150/150 | 152/158 | 251/253 | 174/174 | 209/209 | 180/180 | 172/172 | 237/237 | 187/187 | 148/148 | 199/199 | 266/270 | 254/254 | 334/334 | ND |
| IIa | A1 | Pc51265 | 146/146 | 152/158 | 251/259 | 174/174 | 209/209 | 180/180 | 172/172 | 237/237 | 187/187 | 164/164 | 199/199 | 266/266 | 252/260 | 330/344 | 3.4.6.8 |
| IIa | A1 | Pd11228 | 148/148 | 152/158 | 253/261 | 174/176 | 209/209 | 180/180 | 172/172 | 237/237 | 187/187 | 164/164 | 199/207 | 264/274 | 254/254 | 330/344 | ND |
| Ia | SF | Pd21410 | 148/148 | 150/158 | 253/253 | 172/176 | 209/209 | 182/182 | 168/168 | 237/237 | 187/187 | 180/180 | 199/205 | 266/266 | 254/254 | 338/346 | 1.2.3.4.5.6.7.8.9.10.11 |
| IIb | A1 | F48 | 148/148 | 154/158 | 259/259 | 182/182 | 209/209 | 180/180 | 172/172 | 240/240 | 187/187 | 156/156 | 185/203 | 266/266 | 254/254 | 328/338 | 2.3.4.5.6.7.8.9.10.11 |
| Ia | SF | G29 | 148/148 | 154/154 | 255/255 | 180/182 | 209/209 | 182/182 | 168/168 | 243/243 | 187/187 | 180/180 | 207/215 | 266/266 | 254/254 | 338/346 | 1.2.3.4.5.6.7.8.9.10.11 |

**Supplementary Table 3. Normalized RPKM values of assembled *P*. *infestans* RXLR genes.**

| Gene_id | Sample | | | | |
| --- | --- | --- | --- | --- | --- |
|  | 80029 | F48 | Pa21106 | Pc51265 | Pd21410 |
| PITG_00366 | 36.6420896 | 0 | 53.17281227 | 37.8964118 | 0 |
| PITG_00582 | 119.995504 | 364.23578 | 274.2705762 | 70.8605658 | 371.916201 |
| PITG_00707 | 0 | 109.67342 | 52.4626277 | 0 | 0 |
| PITG_00774 | 77.2629707 | 39.083875 | 195.4988833 | 53.2508282 | 0 |
| PITG_00821 | 410.066796 | 573.14836 | 926.3216539 | 307.134271 | 402.354406 |
| PITG_02830 | 0 | 0 | 136.2668361 | 221.647329 | 0 |
| PITG_02843 | 152.603399 | 57.83797 | 176.3439339 | 78.9187243 | 0 |
| PITG_02860 | 0 | 154.48208 | 464.9410085 | 70.2255365 | 86.6328089 |
| PITG_02897 | 0 | 0 | 4.585511277 | 0 | 0 |
| PITG_02900 | 0 | 80.406492 | 0 | 0 | 67.6106461 |
| PITG_03155 | 28.3210018 | 0 | 16.54285707 | 0 | 0 |
| PITG_03192 | 0 | 69.598144 | 68.29154385 | 158.126839 | 39.0234061 |
| PITG_04049 | 668.725441 | 202.9534 | 774.2465583 | 470.128564 | 341.315307 |
| PITG_04050 | 0 | 0 | 69.75862587 | 0 | 73.8744875 |
| PITG_04052 | 0 | 0 | 22.73783151 | 11.1058487 | 13.7222148 |
| PITG_04063 | 10.7505835 | 16.313341 | 3.248261644 | 0 | 6.86110739 |
| PITG_04074 | 22.7871334 | 0 | 0 | 0 | 0 |
| PITG_04085 | 49.276789 | 0 | 15.83870127 | 0 | 0 |
| PITG_04086 | 49.276789 | 0 | 15.83870127 | 0 | 0 |
| PITG_04089 | 0 | 42.970091 | 404.3851955 | 308.488255 | 159.250771 |
| PITG_04090 | 59.1321468 | 0 | 101.9759861 | 30.6165027 | 75.4229882 |
| PITG_04099 | 0 | 28.296046 | 0 | 0 | 0 |
| PITG_04145 | 74.2633669 | 56.297676 | 274.4117426 | 57.6053189 | 284.193999 |
| PITG_04153 | 0 | 0 | 0 | 39.7270622 | 0 |
| PITG_04169 | 0 | 63.005053 | 0 | 0 | 0 |
| PITG_04196 | 479.956997 | 630.93915 | 765.6462347 | 363.948591 | 898.085803 |
| PITG_04203 | 0 | 0 | 14.15280172 | 27.9244388 | 0 |
| PITG_04266 | 327.308052 | 372.49394 | 239.4392167 | 225.596374 | 243.621903 |
| PITG_04314 | 82.6404675 | 83.597515 | 212.987467 | 113.920419 | 70.2977986 |
| PITG_04339 | 89.063759 | 45.054669 | 88.67622428 | 92.028406 | 37.8906133 |
| PITG_04353 | 0 | 0 | 1.386685423 | 0 | 0 |
| PITG_04388 | 55.0354469 | 16.702691 | 119.7492073 | 34.1129911 | 56.1981648 |
| PITG_05014 | 17.5890958 | 20.014253 | 19.27348207 | 31.7866169 | 0 |
| PITG_05750 | 350.775677 | 228.14915 | 632.5662378 | 543.948232 | 127.885708 |
| PITG_05846 | 119.49718 | 20.149133 | 241.1354966 | 68.5910282 | 321.985298 |
| PITG_05910 | 47.898416 | 123.5896 | 205.9037471 | 128.712894 | 97.8026258 |
| PITG_05911 | 282.953876 | 969.80417 | 458.5331677 | 52.6691524 | 837.450486 |
| PITG_05912 | 28.2953876 | 171.64676 | 95.22349502 | 17.5563841 | 187.704419 |
| PITG_05918 | 11.318155 | 17.164676 | 14.45489674 | 0 | 14.4388015 |
| PITG_05980 | 0 | 19.541938 | 0 | 0 | 0 |
| PITG_05983 | 0 | 21.540246 | 0 | 0 | 0 |
| PITG_06087 | 376.344136 | 171.17261 | 511.7404224 | 389.243468 | 336.045743 |
| PITG_06092 | 0 | 0 | 53.27260323 | 0 | 0 |
| PITG_06094 | 76.5512088 | 116.17323 | 474.1446874 | 263.799396 | 293.042707 |
| PITG_06099 | 382.756044 | 425.9685 | 303.8532856 | 26.3799396 | 748.886917 |
| PITG_06246 | 17.6126935 | 0 | 37.88109286 | 0 | 67.4508243 |
| PITG_06308 | 179.495136 | 83.814066 | 172.0130416 | 42.7982336 | 123.358605 |
| PITG_06375 | 0 | 0 | 9.485342886 | 6.89738698 | 0 |
| PITG_06478 | 63.7087084 | 125.66098 | 226.1686095 | 118.435747 | 48.7961274 |
| PITG_07387 | 0 | 17.990853 | 7.682081459 | 0 | 0 |
| PITG_07414 | 0 | 0 | 31.53040283 | 0 | 0 |
| PITG_07451 | 188.172068 | 285.28768 | 188.7974374 | 38.9243468 | 192.026139 |
| PITG_07533 | 13.4713245 | 0 | 8.738921492 | 0 | 0 |
| PITG_07550 | 585.190953 | 637.60688 | 804.8818795 | 573.666616 | 344.711511 |
| PITG_07555 | 23.7652109 | 468.90165 | 3.105904791 | 0 | 242.604494 |
| PITG_07558 | 87.1933582 | 0 | 60.37898895 | 60.0627647 | 111.285439 |
| PITG_07566 | 0 | 12.849192 | 1.101116504 | 8.74909684 | 0 |
| PITG_07569 | 11.8557111 | 26.98628 | 54.54277836 | 18.3723999 | 105.927065 |
| PITG_07594 | 14.493635 | 131.93804 | 53.2380513 | 29.9706973 | 166.434963 |
| PITG_07597 | 0 | 0 | 3.80271795 | 29.9706973 | 0 |
| PITG_07630 | 31.2798362 | 15.821593 | 60.74332309 | 16.156459 | 66.5439513 |
| PITG_07741 | 89.8413787 | 0 | 137.097273 | 139.431834 | 0 |
| PITG_07947 | 0 | 0 | 3.646934527 | 0 | 0 |
| PITG_08074 | 29.2178515 | 66.511501 | 9.484524892 | 15.0950081 | 18.6454327 |
| PITG_08133 | 70.6888491 | 35.762729 | 218.6110249 | 73.0749421 | 180.405262 |
| PITG_08174 | 77.1716367 | 234.26164 | 133.2121866 | 99.7007861 | 172.347039 |
| PITG_08624 | 9.76453462 | 0 | 18.953204 | 15.1299731 | 12.4638851 |
| PITG_08943 | 574.712367 | 971.35682 | 1640.663302 | 1135.84816 | 84.5839338 |
| PITG_09160 | 701.228756 | 697.27291 | 919.8767252 | 674.918373 | 1079.92887 |
| PITG_09216 | 1993.87417 | 1625.5592 | 2398.667047 | 1107.11981 | 1715.59797 |
| PITG_09218 | 881.492183 | 339.15985 | 2270.09702 | 2053.59117 | 1108.97062 |
| PITG_09223 | 61.8540363 | 46.914787 | 207.2109336 | 191.870445 | 0 |
| PITG_09224 | 291.333802 | 392.7873 | 600.2498415 | 301.254222 | 454.276153 |
| PITG_09316 | 25.3576128 | 12.825385 | 18.5769276 | 26.1921588 | 53.9475741 |
| PITG_09585 | 9.69612436 | 29.425672 | 58.96779586 | 30.0478283 | 61.8829118 |
| PITG_09622 | 12.3862394 | 0 | 0 | 0 | 0 |
| PITG_09647 | 24.7724788 | 93.980985 | 104.3751264 | 76.7804858 | 15.8094055 |
| PITG_09732 | 45.9716372 | 59.786597 | 145.1632285 | 61.0450978 | 117.368982 |
| PITG_09836 | 0 | 0 | 0 | 0 | 33.1711829 |
| PITG_09861 | 15.42293 | 23.40636 | 6.01126505 | 15.9368351 | 0 |
| PITG_09935 | 0 | 0 | 0 | 0 | 45.5414592 |
| PITG_10116 | 0 | 0 | 6.774966677 | 32.4351707 | 0 |
| PITG_10232 | 23.1685617 | 0 | 10.08881988 | 0 | 44.346688 |
| PITG_10396 | 0 | 0 | 10.15739871 | 0 | 0 |
| PITG_10540 | 79.890104 | 48.487601 | 158.4883321 | 82.5171963 | 137.67216 |
| PITG_10654 | 194.763748 | 337.73986 | 273.5200895 | 115.06513 | 319.514181 |
| PITG_10672 | 32.3704224 | 0 | 17.0283643 | 33.4726914 | 41.2978321 |
| PITG_10808 | 0 | 0 | 25.9449353 | 0 | 0 |
| PITG_10818 | 20.3923283 | 0 | 19.79703862 | 10.5327756 | 13.0147087 |
| PITG_10835 | 103.353455 | 67.22136 | 182.148584 | 91.5370965 | 113.065696 |
| PITG_11484 | 475.362511 | 823.90443 | 460.4704219 | 0 | 0 |
| PITG_11947 | 159.300988 | 114.49588 | 383.6940615 | 381.047211 | 128.428554 |
| PITG_12706 | 171.398198 | 0 | 8.516677256 | 59.1059545 | 0 |
| PITG_12710 | 295.660734 | 0 | 39.59675318 | 20.4110018 | 75.4229882 |
| PITG_12731 | 148.219336 | 0 | 125.5164232 | 126.082116 | 77.9051497 |
| PITG_12737 | 169.262114 | 256.89002 | 622.7344154 | 224.972791 | 431.971547 |
| PITG_12791 | 0 | 0 | 5.824348039 | 0 | 0 |
| PITG_13044 | 56.0683263 | 74.451894 | 74.03516018 | 50.690443 | 62.6160483 |
| PITG_13045 | 16.9772325 | 51.494027 | 86.95290977 | 122.894689 | 21.6582022 |
| PITG_13047 | 10.526941 | 31.956714 | 42.43009582 | 54.405967 | 40.300847 |
| PITG_13048 | 168.204979 | 95.723863 | 114.059629 | 36.2074593 | 98.3966473 |
| PITG_13093 | 440.74916 | 376.18882 | 333.3193692 | 170.880629 | 175.744497 |
| PITG_13452 | 283.892397 | 2006.0193 | 2543.996747 | 764.568291 | 563.296169 |
| PITG_13503 | 0 | 0 | 1.82864631 | 29.1135177 | 0 |
| PITG_13507 | 0 | 0 | 0 | 49.2039593 | 0 |
| PITG_13509 | 33.4556524 | 0 | 8.701197049 | 0 | 21.3487685 |
| PITG_13529 | 51.0341392 | 0 | 93.49331865 | 0 | 65.1206015 |
| PITG_13628 | 29.5295587 | 22.40712 | 15.33882813 | 0 | 18.8442826 |
| PITG_13847 | 121.011311 | 119.34213 | 185.8556743 | 174.967581 | 15.4477213 |
| PITG_13956 | 0 | 0 | 3.455123855 | 7.8732607 | 0 |
| PITG_14054 | 0 | 0 | 6.149829615 | 0 | 0 |
| PITG_14093 | 71.4008415 | 0 | 9.412908572 | 0 | 0 |
| PITG_14360 | 1923.70527 | 907.35098 | 2130.21379 | 2072.39864 | 497.567743 |
| PITG_14371 | 1865.19996 | 224.58164 | 1529.020533 | 520.393763 | 1756.52593 |
| PITG_14374 | 0 | 0 | 5.828540788 | 0 | 18.8873756 |
| PITG_14443 | 0 | 0 | 16.14211181 | 0 | 44.346688 |
| PITG_14662 | 34.374845 | 0 | 95.07381339 | 17.7740481 | 43.852128 |
| PITG_14685 | 26.6488775 | 151.62541 | 107.5273337 | 89.4501305 | 85.0455053 |
| PITG_14783 | 669.055539 | 141.30998 | 1053.64686 | 785.450216 | 2702.66945 |
| PITG_14787 | 632.890374 | 141.30998 | 1559.195818 | 1234.27891 | 4732.5549 |
| PITG_14788 | 0 | 0 | 5.19697743 | 0 | 0 |
| PITG_14884 | 45.3374506 | 38.214385 | 47.75240119 | 26.0113653 | 38.5846241 |
| PITG_14932 | 21.8940924 | 31.082422 | 106.9768941 | 36.0696501 | 13.9682546 |
| PITG_14954 | 431.493675 | 182.81434 | 946.8556926 | 643.65211 | 249.63155 |
| PITG_14955 | 107.452183 | 41.497733 | 202.3535692 | 177.957529 | 24.8952588 |
| PITG_14959 | 431.493675 | 182.81434 | 944.8529842 | 617.98222 | 249.63155 |
| PITG_14960 | 256.791502 | 124.04457 | 567.1111524 | 363.178006 | 184.375796 |
| PITG_14961 | 431.493675 | 182.81434 | 942.5255123 | 617.98222 | 249.63155 |
| PITG_14962 | 431.493675 | 182.81434 | 599.0880876 | 617.98222 | 249.63155 |
| PITG_14965 | 213.467401 | 108.44312 | 437.0378525 | 245.298844 | 30.2645517 |
| PITG_15038 | 6.26985354 | 0 | 8.093331712 | 0 | 8.00375335 |
| PITG_15039 | 18.3929313 | 0 | 49.06240863 | 75.9830132 | 31.3059836 |
| PITG_15105 | 0 | 0 | 0.5579755 | 4.46991669 | 0 |
| PITG_15110 | 25.3371048 | 3.2033784 | 28.84868963 | 15.2627298 | 53.9091145 |
| PITG_15114 | 11.8422667 | 0 | 38.20658098 | 12.2302469 | 37.7932796 |
| PITG_15123 | 30.9902842 | 9.1005771 | 42.3990424 | 8.25954535 | 42.1130583 |
| PITG_15125 | 18.9940451 | 22.751443 | 31.9485742 | 17.5515339 | 45.9415181 |
| PITG_15127 | 9.99686586 | 4.5502886 | 14.53112721 | 5.16221584 | 3.82845984 |
| PITG_15142 | 19.0250344 | 9.6219288 | 29.47125315 | 26.1984118 | 48.5725651 |
| PITG_15152 | 0 | 35.665221 | 29.38591561 | 32.3672438 | 0 |
| PITG_15177 | 0 | 36.698574 | 11.06380941 | 0 | 30.8551105 |
| PITG_15235 | 106.895571 | 0 | 122.7314242 | 154.692577 | 81.8461938 |
| PITG_15278 | 475.72599 | 288.71548 | 352.2497423 | 399.191481 | 781.886294 |
| PITG_15287 | 34.7144098 | 0 | 33.87228313 | 0 | 0 |
| PITG_15297 | 0 | 63.956621 | 69.86564781 | 163.712134 | 0 |
| PITG_15315 | 0 | 0 | 9.054648894 | 0 | 0 |
| PITG_15318 | 0 | 127.91324 | 47.50864051 | 76.398996 | 0 |
| PITG_15454 | 171.398198 | 0 | 8.516677256 | 59.1059545 | 0 |
| PITG_15679 | 25.4559521 | 0 | 14.85425944 | 0 | 0 |
| PITG_15753 | 0 | 37.684428 | 77.94324533 | 77.0096689 | 31.6848749 |
| PITG_15930 | 43.1436557 | 43.649589 | 51.34352046 | 14.8595319 | 36.7098076 |
| PITG_15940 | 0 | 0 | 2.292755638 | 0 | 0 |
| PITG_16195 | 91.1570099 | 27.661443 | 109.024633 | 43.9340353 | 46.5466319 |
| PITG_16233 | 590.189275 | 229.25655 | 32.57292143 | 44.5855445 | 221.619113 |
| PITG_16235 | 73.2841792 | 55.557659 | 64.29076392 | 31.5803432 | 15.580541 |
| PITG_16240 | 590.189275 | 229.25655 | 32.57292143 | 44.5855445 | 221.619113 |
| PITG_16242 | 183.210448 | 0 | 237.3901355 | 214.746334 | 186.966492 |
| PITG_16275 | 1093.1997 | 324.62422 | 1602.646872 | 712.459162 | 818.790168 |
| PITG_16294 | 250.410533 | 105.54371 | 449.428497 | 290.060015 | 497.022059 |
| PITG_16409 | 91.605224 | 27.778829 | 42.99862048 | 56.8446178 | 15.580541 |
| PITG_16424 | 128.247314 | 27.778829 | 97.37990575 | 37.8964118 | 62.322164 |
| PITG_16427 | 649.871337 | 133.87889 | 43.83973274 | 70.307974 | 456.772218 |
| PITG_16663 | 197.109399 | 0 | 0 | 0 | 0 |
| PITG_16705 | 438.815719 | 238.73185 | 426.5650691 | 393.648903 | 523.411319 |
| PITG_16726 | 6.09050591 | 13.861159 | 2.751290969 | 0 | 3.88741978 |
| PITG_16737 | 44.0593172 | 22.288182 | 85.18265805 | 15.1751675 | 37.4886606 |
| PITG_16844 | 5.41737336 | 0 | 4.910828937 | 5.59642338 | 20.7444235 |
| PITG_16845 | 16.2521201 | 0 | 11.92629885 | 5.59642338 | 20.7444235 |
| PITG_17063 | 389.940257 | 118.35013 | 462.7940503 | 430.007786 | 331.711829 |
| PITG_17218 | 0 | 48.940023 | 21.34571937 | 0 | 0 |
| PITG_17309 | 103.047614 | 113.71808 | 134.6761907 | 94.348591 | 80.7151053 |
| PITG_17316 | 74.9437191 | 99.50332 | 150.4307731 | 74.9950339 | 116.588485 |
| PITG_17670 | 0 | 0 | 7.782927211 | 0 | 0 |
| PITG_18156 | 35.3799898 | 0 | 18.41593129 | 0 | 0 |
| PITG_18215 | 878.806584 | 1155.516 | 3.843759363 | 0 | 0 |
| PITG_18325 | 1905.38866 | 1388.9415 | 1358.323659 | 757.928237 | 1495.73194 |
| PITG_18609 | 43.4809049 | 43.979346 | 30.4217436 | 0 | 55.4783209 |
| PITG_18670 | 71.3508564 | 178.29814 | 105.5732035 | 198.497799 | 56.8781207 |
| PITG_18683 | 19.7107156 | 0 | 0 | 0 | 0 |
| PITG_18685 | 124.863999 | 16.208922 | 234.3558485 | 87.7083297 | 104.655742 |
| PITG_18981 | 0 | 46.914787 | 0 | 0 | 0 |
| PITG_19302 | 0 | 0 | 0 | 6.30398155 | 0 |
| PITG_19307 | 7.85301562 | 0 | 7.103768483 | 0 | 10.0243719 |
| PITG_19617 | 0 | 0 | 0 | 0 | 54.6565009 |
| PITG_19655 | 0 | 0 | 20.28320934 | 14.0858838 | 11.604399 |
| PITG_19942 | 0 | 0 | 164.9396495 | 88.6589317 | 163.969503 |
| PITG_19992 | 0 | 0 | 26.16240045 | 14.0858838 | 23.2087979 |
| PITG_19994 | 0 | 20.543454 | 3.512828137 | 0 | 0 |
| PITG_20144 | 0 | 186.75004 | 30.57352021 | 0 | 157.016966 |
| PITG_20300 | 0 | 0 | 43.90074809 | 30.6165027 | 0 |
| PITG_20301 | 0 | 0 | 2.639783546 | 0 | 0 |
| PITG_20303 | 0 | 0 | 2.639783546 | 0 | 0 |
| PITG_20336 | 163.501869 | 133.96628 | 13.05213503 | 53.9945877 | 94.872449 |
| PITG_20616 | 0 | 0 | 13.10752612 | 4.75783337 | 11.7588501 |
| PITG_20934 | 261.937937 | 168.94477 | 6.707479109 | 24.0076009 | 179.325389 |
| PITG_20936 | 261.937937 | 168.94477 | 6.707479109 | 24.0076009 | 179.325389 |
| PITG_21190 | 0 | 0 | 6.273169772 | 0 | 0 |
| PITG_21238 | 0 | 28.528768 | 49.68353616 | 77.8486937 | 0 |
| PITG_21362 | 29.3210201 | 44.497633 | 63.77302948 | 30.2967066 | 65.4893681 |
| PITG_21388 | 1180.43311 | 2046.9428 | 2494.524994 | 1540.08725 | 1434.45411 |
| PITG_21645 | 0 | 0 | 3.455123855 | 7.8732607 | 0 |
| PITG_21740 | 4.2228508 | 16.016892 | 10.34198307 | 10.9019499 | 32.3454687 |
| PITG_21949 | 0 | 0 | 1.723317303 | 0 | 0 |
| PITG_22089 | 662.112069 | 2548.9543 | 692.4938675 | 175.563841 | 1646.02337 |
| PITG_22375 | 0 | 0 | 2.031479742 | 0 | 0 |
| PITG_22547 | 223.979932 | 121.33445 | 392.2237148 | 333.619542 | 326.576656 |
| PITG_22604 | 124.863999 | 16.208922 | 234.3558485 | 87.7083297 | 104.655742 |
| PITG_22675 | 0 | 63.005053 | 379.7345227 | 172.014323 | 212.102579 |
| PITG_22712 | 30.0339023 | 0 | 7.886089897 | 0 | 114.95924 |
| PITG_22722 | 0 | 0 | 1.392112133 | 0 | 6.86110739 |
| PITG_22724 | 26.7490176 | 0 | 100.430172 | 9.21820713 | 0 |
| PITG_22725 | 0 | 0 | 13.44912878 | 0 | 0 |
| PITG_22740 | 5.30797963 | 0 | 0 | 0 | 13.5520507 |
| PITG_22757 | 618.103765 | 78.16775 | 1392.086876 | 266.254141 | 295.766229 |
| PITG_22798 | 237.652109 | 0 | 220.5192402 | 171.972901 | 30.3255618 |
| PITG_22804 | 527.28395 | 266.65753 | 395.9072144 | 181.726012 | 261.632004 |
| PITG_22825 | 0 | 44.916329 | 120.4565096 | 183.668387 | 37.7747512 |
| PITG_22828 | 33.9544651 | 51.494027 | 62.58821269 | 175.563841 | 43.3164044 |
| PITG_22870 | 574.712367 | 971.35682 | 1640.663302 | 1135.84816 | 84.5839338 |
| PITG_22880 | 18.7696048 | 0 | 0 | 0 | 0 |
| PITG_22884 | 0 | 0 | 3.609957067 | 0 | 0 |
| PITG_22922 | 6.37087084 | 38.664917 | 21.38321399 | 0 | 8.1326879 |
| PITG_22926 | 47.898416 | 36.349883 | 63.64297638 | 29.7029756 | 48.9013129 |
| PITG_23008 | 0 | 0 | 4.753978768 | 0 | 0 |
| PITG_23009 | 0 | 0 | 4.753978768 | 0 | 0 |
| PITG_23011 | 0 | 0 | 0 | 0 | 480.051661 |
| PITG_23014 | 542.143315 | 400.19386 | 792.1071992 | 171.397877 | 609.211927 |
| PITG_23015 | 542.143315 | 400.19386 | 798.3737072 | 171.397877 | 609.211927 |
| PITG_23035 | 0 | 19.07759 | 14.57677985 | 9.73861079 | 8.02677445 |
| PITG_23036 | 6.43007006 | 19.512131 | 9.961296974 | 0 | 0 |
| PITG_23042 | 99.4308862 | 201.07131 | 130.8373652 | 171.366024 | 126.849455 |
| PITG_23046 | 24.0051928 | 0 | 67.03181802 | 24.8409215 | 0 |
| PITG_23092 | 47.1258994 | 35.762729 | 110.8450267 | 73.0749421 | 300.675436 |
| PITG_23129 | 48.8492049 | 73.99874 | 152.7294271 | 25.2760835 | 62.305475 |
| PITG_23131 | 4093.44913 | 2302.4384 | 5737.366998 | 3296.07097 | 2906.76033 |
| PITG_23135 | 0 | 0 | 13.44912878 | 0 | 0 |
| PITG_23137 | 0 | 0 | 28.61092108 | 23.0455178 | 0 |
| PITG_23185 | 0 | 0 | 2.823167702 | 0 | 0 |
| PITG_23202 | 16.7720954 | 0 | 11.03811084 | 17.3439626 | 0 |
| PITG_23206 | 0 | 0 | 41.17253136 | 0 | 128.08544 |
| PITG_23226 | 141.344659 | 156.21814 | 228.3771943 | 56.7368149 | 201.388826 |
| PITG_23230 | 120.444436 | 173.6947 | 21.60267009 | 87.8041986 | 190.956334 |
| PITG_23239 | 0 | 0 | 28.61092108 | 23.0455178 | 0 |

**Supplementary Table 4. Detection of a novel RXLR effector gene.**

| Gene_ID | comp858_c1_seq1 |
| --- | --- |
| NCBI number | MG269997 |
| Nucleotide sequence | CGATCTCAACGCGCAAGCACGCTACATAGGACCATGCGCGCTTCGCCCATCGCCATAGCTTTTCTCATGGCCATCTCCACAGCAAGTGAAGCCACTGTCGCTAACTCTGATCGTGCTAGGAAATCCAACGCTGATGACAGTATGAGGAACAACCACCGATTCCTGAGGGACAACGAAATTGATATCGAAGCCGAGGATCGAATGGCCTGGTCCTTTAACTTTAAAAAAGATGGACTTGCGGACAGGATTATAAGGGCCACGCAGTGGGATTCAAAGGTCGCGATTCTCAAGGATATGAAGGAAGGTCAGATGAACTACGCGTTCGACGACTTGGTACGAACCATACAATTGTTCATCCCCAGTTACGAAGCAGGCATGAACAAGGGCGCGTTTAT |
| Amino acid sequence | MRASPIAIAFLMAISTASEATVANSDRARKSNADDSMRNNHRFLRDNEIDIEAEDRMAWSFNFKKDGLADRIIRATQWDSKVAILKDMKEGQMNYAFDDLVRTIQLFIPSYEAGMNKGAF |
