# Supplementary material for: Conserved RXLR Effector Genes of Phytophthora infestans Expressed at the Early Stage of Potato Infection Are Suppressive to Host Defense
Source: Front Plant Sci. 2017 Dec 19;8:2155. doi: 10.3389/fpls.2017.02155 (PMC5742156; doi:10.3389/fpls.2017.02155)
Supplement: Supplementary file 2 [file Image_1.PDF]

Supplementary Material

Conserved RXLR effector genes of *Phytophthora infestans* expressed at the early stage of potato infection are suppressive to host defense

Conserved RXLR effector genes of *Phytophthora infestans* expressed at the early stage of potato infection are suppressive to host defense

Junliang Yin[1,3], Biao Gu[1,3], Guiyan Huang[2,3], Yuee Tian[1,5], Junli Quan[1,3], Hannele Lindqvist-Kreuze[4] and Weixing Shan[1,3]

[1]College of Plant Protection, Northwest A&F University, Yangling, Shaanxi 712100, China

[2]College of Life Sciences, Northwest A&F University, Yangling, Shaanxi 712100, China

[3]State Key Laboratory of Crop Stress Biology for Arid Areas, Northwest A&F University, Yangling, Shaanxi 712100, China

[4]International Potato Center (CIP), Lima, Peru

[5]Present address: College of Forestry, Henan University of Science and Technology, Luoyang, Henan 471023, China

*** Correspondence:**
Weixing Shan, College of Agronomy, Northwest A&F University, 3 Taicheng Road, Yangling, Shaanxi 712100, China
wxshan@nwafu.edu.cn

Supplementary Figures

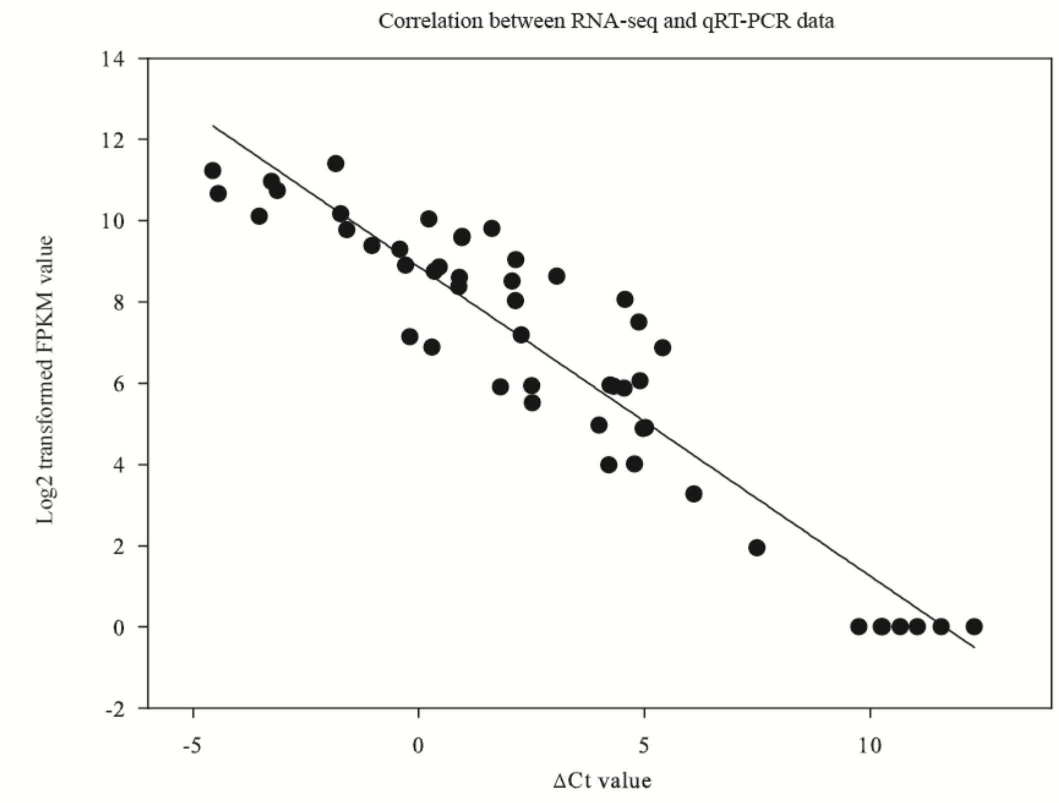

Supplementary Figure 1. RNA-seq sequencing data confirmation by qRT-PCR. ΔCt values were calculated by the Ct values minus the Ct values of ten randomly selected reference RXLR effector genes. Totally, five samples produced fifty points that were draw in the figure. Significant negative correlation was found existence between the ΔCt values from qRT-PCR and the values of log2 transformed expression levels from RNA-seq sequencing data ($R^2 = 0.8783$, *p*-value < 2.2e-16).

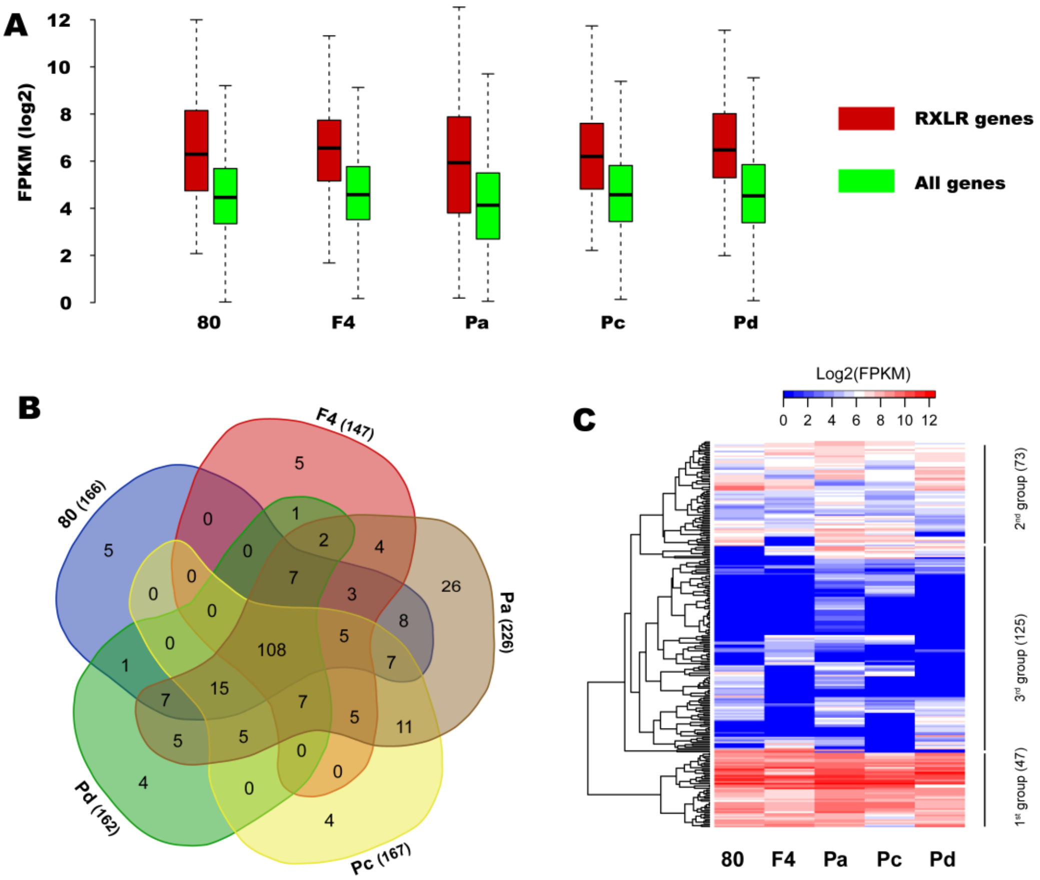

Supplementary Figure 2. ***P. infestans*** **RXLR effector genes expressed at early stage (12 hpi) of potato infection.** (**A)** Boxplot overviews indicating higher expression levels of RXLR effector genes (red bar) compared to all assembled *P. infestans* genes in the transcriptome datasets (green bar). (**B)** Venn diagram showing the number and distribution of expressed RXLR effector genes in the examined *P. infestans* strains. (**C)** Heatmap overviews indicating the number and expression levels of RXLR effector genes detected in each strain. Strain names: Pa, Pa21106; Pc, Pc51265; Pd, Pd21410; F4, F48; 80, 80029.

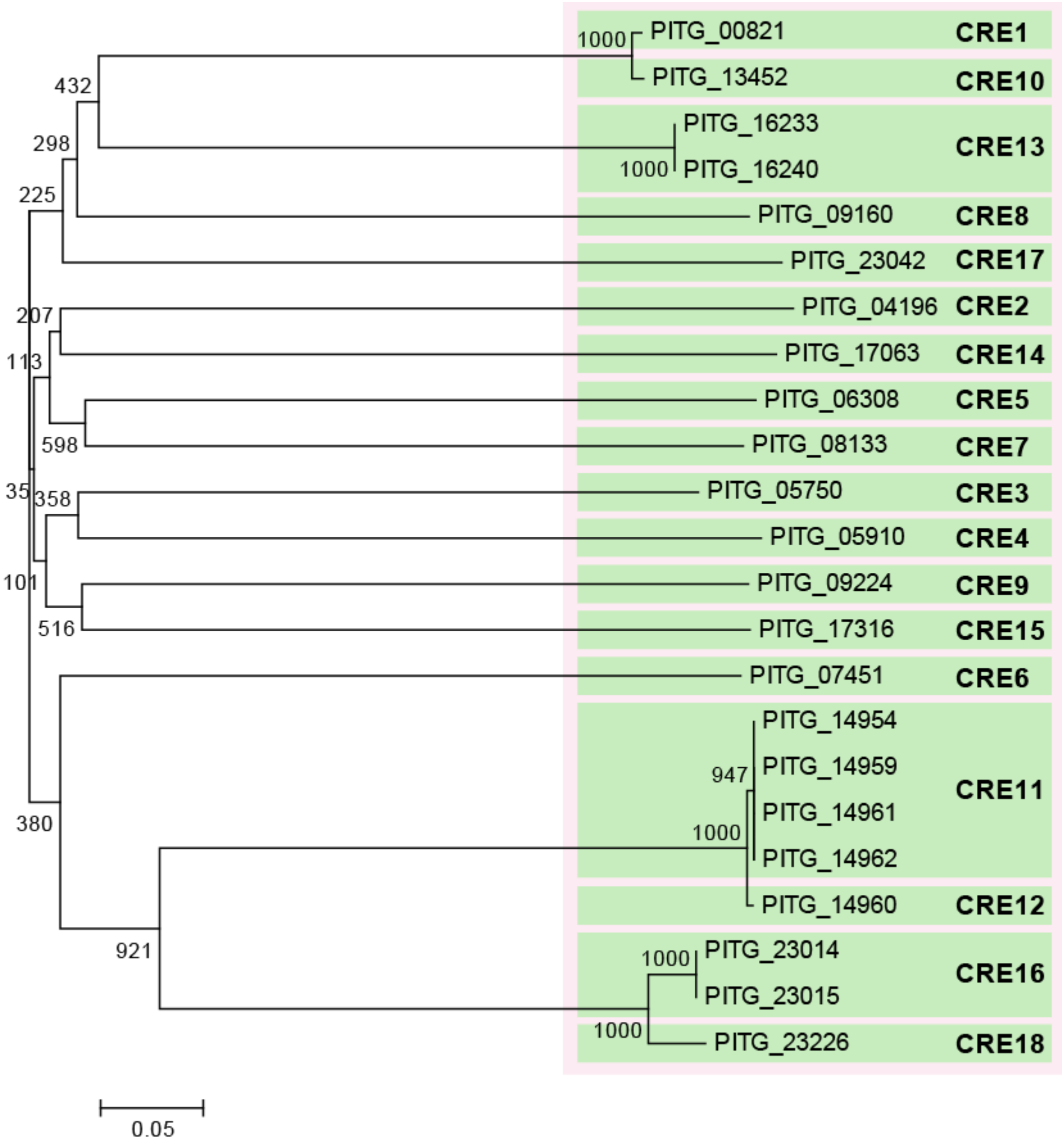

Supplementary Figure 3. Sequence phylogenetic analysis of 18 core RXLR effectors. The phylogenetic tree was constructed by MEGA6.0 (Bootstrap value = 1000). PITG_14954, PITG_14959, PITG_14961 and PITG_14962, designated as CRE11 (Core RXLR Effector), have same nucleotide sequence. Members of CRE13 (PITG_16233 and PITG_16240) and CRE16 (PITG_23014 and PITG_23015) are also with same nucleotide sequence.

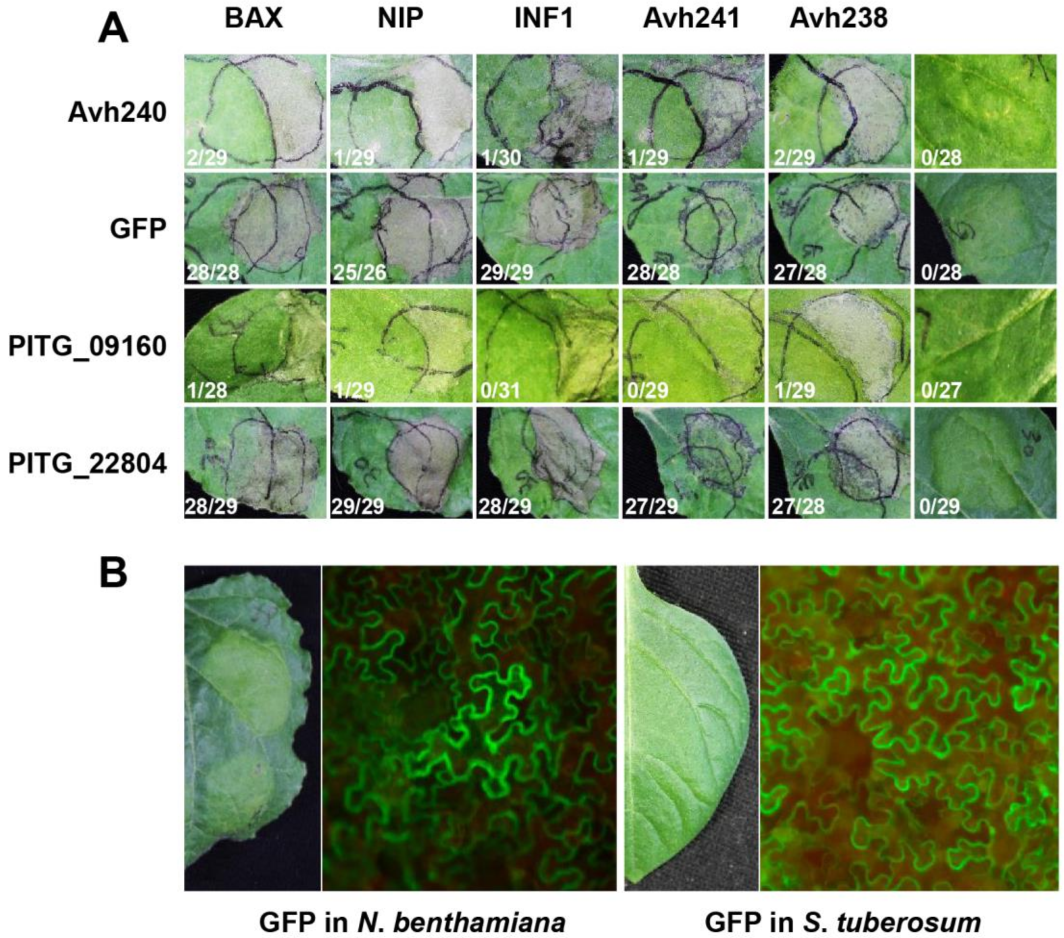

Supplementary Figure 4. Plant defense suppression activities of selected *P. infestans* RXLR effector genes. *A. tumefaciens*-mediated transient expression assays of andidate RXLR effector gene PITG_09160 (CRE8) were performed on *N. benthamiana* (**A)**. The elicitors including: BAX; the *P. infestans* PAMP elicitor INF1; NIP; the *P. sojae* RXLR effectors Avh238 and Avh241. The positive control Avh240, and the negative control GFP and PITG_22804. **(B)** Confirmation of the transient expression assays in *N. benthamiana* and *S. tuberosum*.

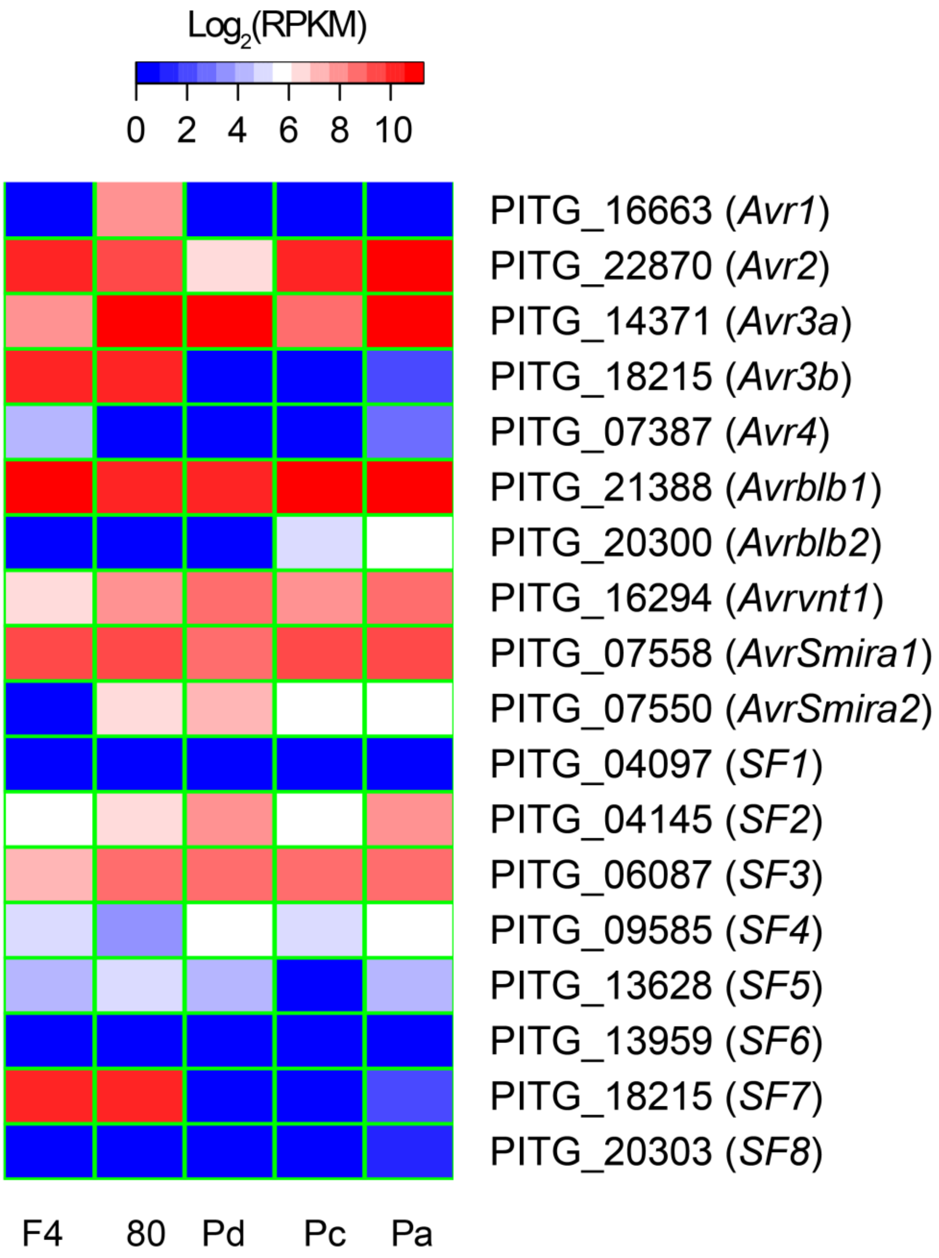

Supplementary Figure 5. Expression profiles of known avirulence and virulence RXLR effector genes in the examined *P. infestans* strains. Eighteen RXLR effectors, including ten known avirulence genes and eight SFI (Suppressor of early Flg22-induced Immune response) virulence genes were included in the heatmap generated using R package “gplots”. Strain names: F4, F48; 80, 80029; Pd, Pd21410; Pc, Pc51265; Pa, Pa2110
